# A highly sensitive and multiplexed wireless sensing system with skin-like compliance and stretchability for wearable applications

**DOI:** 10.1126/sciadv.adt4923

**Published:** 2025-10-29

**Authors:** Zhilu Ye, Ganggang Zhao, Minye Yang, Yadong Xu, Yichong Ren, Zehua Chen, Syed Muntazir Andrabi, Jingwei Xie, Wei Gao, Zheng Yan, Pai-Yen Chen

**Affiliations:** ^1^ Department of Electrical and Computer Engineering, University of Illinois Chicago, Chicago, IL 60607, USA.; ^2^ Department of Mechanical & Aerospace Engineering, University of Missouri, Columbia, MO 65211, USA.; ^3^ Department of Chemistry and Biochemistry, University of California, Los Angeles, Los Angeles, CA 90095, USA.; ^4^ Department of Chemical and Biomedical Engineering, University of Missouri, Columbia, MO 65211, USA.; ^5^ Andrew and Peggy Cherng Department of Medical Engineering, Division of Engineering and Applied Science, California Institute of Technology, Pasadena, CA 91125, USA.; ^6^ Department of Surgery-Transplant and Mary and Dick Holland Regenerative Medicine Program, University of Nebraska Medical Center, Omaha, NE 68130, USA.

## Abstract

Wearable wireless sensing systems capable of monitoring critical biomarkers are increasingly valued for health monitoring and disease management. However, current systems often compromise on sensitivity, multi-parameter sensing capacity, compactness, power consumption, and wearing comfort, limiting their skin-interfaced applications. Here, we present a wireless, battery-free, multiplexed sensing system based on a high-order exceptional point (EP)—a singularity in non-Hermitian realm. This system features multiple distinct resonance frequencies that sensitively respond to physiological variations, exhibiting over 10-fold sensitivity improvement and 50% size reduction compared to conventional resonator-based systems. Moreover, the sensor employs a strain-resilient liquid metal composite and a soft substrate, ensuring wearing comfort, mechanical reliability, and antimicrobial properties. The effectiveness of this system is demonstrated through in situ monitoring of skin temperature and sweat electrolytes during exercise and prolonged monitoring of sweat glucose and ammonium in different subjects. The proposed system is highly transformative, offering benefits for various wearable applications.

## Teaser

Singularity-enabled wireless sensing systems exhibit sensitive, multiplexed sensing performance and superior wearing comfort.

## INTRODUCTION

In recent pasts, wearable wireless sensing systems have attracted substantial attention for their capacity to monitor essential biomarkers without the need for cumbersome wire connections to external equipment (*1*–*6*). This advancement allows individuals to conveniently realize self-health status monitoring, eliminating mobility constraints and reducing the necessity for frequent healthcare visits. Numerous wearable wireless sensing systems have been developed for monitoring heart rate (*1*, *7*), respiration rate (*4*, *8*), body temperature (*2*, *9*), blood pressure (*6*, *10*), etc. These systems can be generally categorized into digital and analog types based on their data acquisition mechanisms. The digital systems use Bluetooth or near-field communication (NFC) technologies, converting measured biosignals into digital bitstrings for wireless transmission (*11*–*17*), while the analog ones, exemplified by inductance-capacitance (“LC”) resonators, directly monitor biomarker variations by tracking the frequency or amplitude changes of the spectral resonance (*5*, *18*–*22*).

Both digital and analog systems demonstrate their promises for wearable wireless monitoring, however, facing compromises in sensing performance and wearing comfort (tables S1 and S2). Specifically, digital systems, particularly those capable of simultaneously detecting multiple biomarkers, inevitably comprise numerous rigid and bulky components (e.g., integrated circuit chips and batteries), resulting in poor stretchability, biocompatibility, and maintenance issues (*11*–*17*). Conversely, the analog LC sensing systems may offer a battery-free (i.e., passive) design with minimal rigid components, enabling construction on flexible and stretchable materials for enhanced wearing comfort. Nonetheless, these systems unavoidably suffer from limited sensitivity and sensing capacity, as an LC resonator produces only a single and weak spectral resonance (*5*, *18*–*22*), which impedes the simultaneous detection of multiple minute physiological variations.

These limitations have restricted the use of wireless sensing systems in skin-interfaced applications (e.g., perspiration and wound monitoring), which demand high skin conformality and comfort, alongside high sensitivity and multiplexed sensing capacity to capture subtle variations in multiple biomarkers. Actually, balancing these competing requirements has long posed a challenge in the design of wearable sensing systems. To address this, we focus on the analog regime, taking advantage of its battery-free operation, while directing attention toward an emerging concept—non-Hermitian systems—to achieve enhanced sensing performance (*23*–*28*). Spectral singularities featured by non-Hermitian systems, particularly the exceptional points (EPs), have demonstrated substantial potential for enhanced sensitivity, as they facilitate notable eigenfrequency bifurcation in response to minute perturbations (*25*, *26*, *29*–*32*). The EP sensing systems, realized by parity-time (*PT*)–symmetric circuits, use a reader-sensor scheme where the sensor can be fully passive and constructed using simple circuit configurations, indicating great potential for wearable applications (*25*, *32*–*34*). However, despite these advantages, the EP systems are still constrained by limited sensing capacity, with one sensing resonator only detecting a single parameter. In addition, current EP sensing systems typically use rigid materials. Their integration with soft, stretchable materials, crucial for enhancing comfort and wearability in wearable devices, remains an area requiring further exploration.

**Fig. 1.**
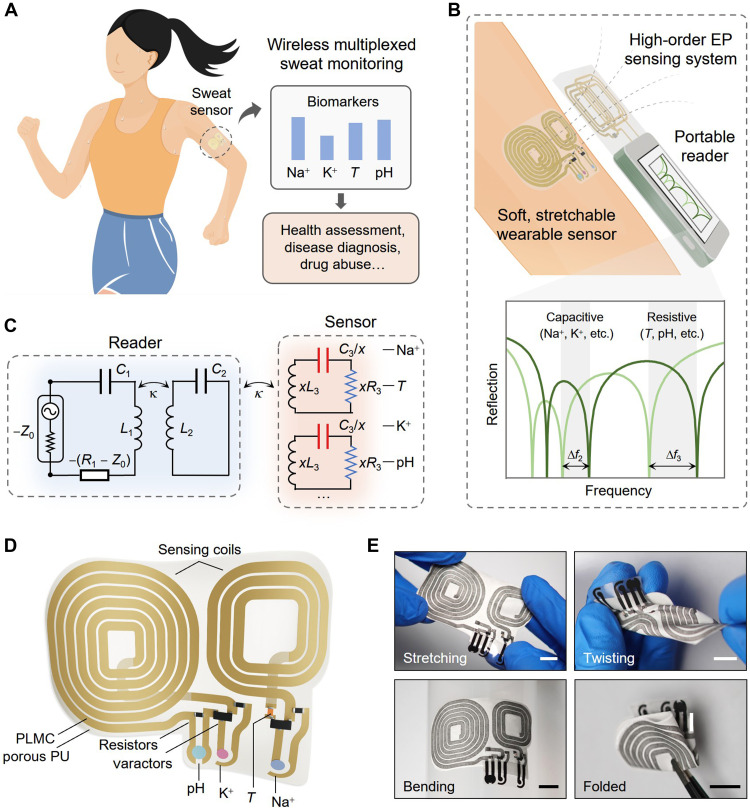
Wireless multiplexed sensing system with an ultrasoft, stretchable, wearable sensor for on-skin perspiration monitoring. (**A**) Schematic illustration of the wireless system for monitoring skin temperature (*T*) and sweat biomarkers (e.g., Na^+^, K^+^, and pH), providing information for health status and disease evaluation. (**B**) Detailed illustration of the wireless sensing system and the sensing mechanism. The soft, stretchable sensor made of PLMC and porous PU is attached and conformal to human skin, communicating with the portable reader through inductive coupling. Resistive and capacitive biomarkers can be simultaneously detected by tracking the resonance frequencies in the reflection spectrum. (**C**) Circuit diagram of the wireless multiplexed sensing system, comprising a reader and a wearable sensor. (**D**) Schematic diagram of the wearable sensor made of PLMC and porous PU, consisting of sensing coils, varactor diodes, resistors, and transducers for biomarkers, e.g., *T*, Na^+^, K^+^, and pH. (**E**) Photographs showing the wearable sensor under mechanical deformations, i.e., stretching, twisting, bending, and folding. Scale bars, 1 cm.

In this work, we present a multiplexed wearable wireless sensing system based on a high-order (i.e., third-order) EP, which enables sensitive monitoring of multiple biomarkers while maintaining passive functionality, stretchability, and tissue-like compliance for skin-interfaced applications (Fig. 1, A and B). The sensitivity of the system is notably improved owing to the enhanced frequency bifurcation at the high-order EP. Concurrently, unlike traditional LC- or EP-based systems limited to single-parameter monitoring, our third-order EP sensing system features multiple distinct resonance frequencies, allowing simultaneous monitoring of two parameters with a single resonator and thus reducing the sensor size by half (Fig. 1, B and C). The wearable sensor is fabricated using a conductive porous liquid metal (LM) composite (PLMC) on a porous polyurethane (PU) substrate. These materials provide reliable conductivity under mechanical deformations, high resistance to LM leakage, and excellent antimicrobial properties, endowing the sensor with superior wearing comfort and biocompatibility (Fig. 1, D and E). Consequently, the proposed wireless sensing system combines the benefits of high sensitivity and multiplexed sensing capability (brought by the high-order EP) and excellent wearing comfort and biocompatibility (gained from the PLMC and porous PU). We demonstrate the capability of the system in on-skin perspiration monitoring, measuring skin temperature and sweat electrolytes (Na^+^, K^+^, and pH) during physical exercise. Further, the system is practically used for prolonged monitoring of sweat NH_4_^+^ and glucose levels in normal and obese volunteers over 10-hour daily activity periods. Given these benefits and capabilities, we are confident that the developed high-order EP-based platform may provide an avenue for health monitoring devices and a variety of wearable biomedical systems.

## RESULTS

### Construction of the wearable sensor and characteristics of materials

Our proposed wearable wireless sensing system enabled by the high-order EP is established in a reader-sensor scheme, where the reader consists of active and neutral resonators, and the wearable sensor comprises fully passive resonators (Fig. 1C and fig. S1). The system design will be explicitly discussed in the following session. Here, we emphasize the construction of the wearable sensor of the system as it directly contacts the skin. As shown in Fig. 1D, the wearable sensor is lightweight and fully flexible, with transducers to translate biomarker variations to resistive or capacitive changes, which are then wirelessly interrogated by the reader. We use PLMC as the conductive material for the top and bottom circuit layers and porous PU as the substrate for sensor construction, endowing the sensor with skin-like compliance and exceptional wearability (Fig. 1, D and E, and fig. S2). In the following, we demonstrate the mechanical resilience, LM leakage prevention, and antibacterial properties of the wearable sensor, which are of paramount importance for ensuring sensor’s practical application.

**Fig. 2.**
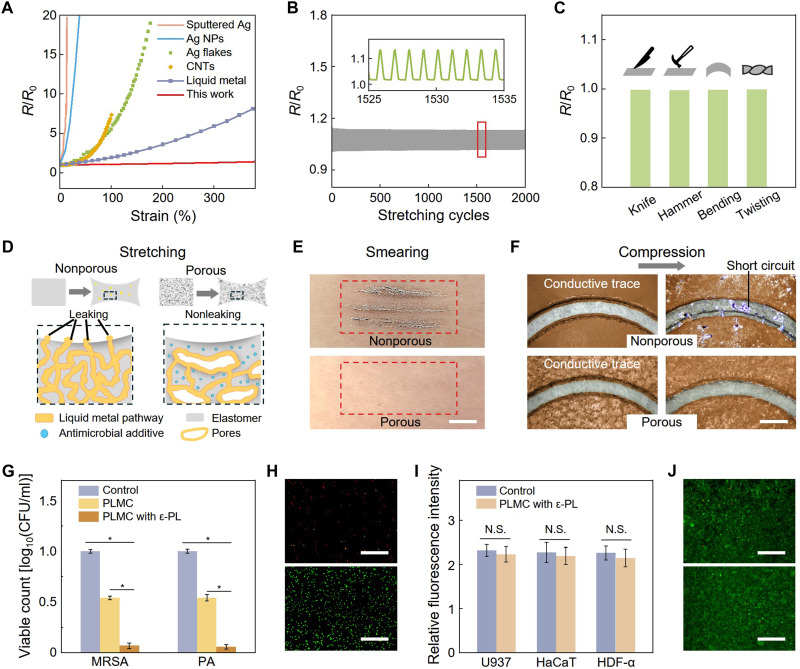
Characterization of the PLMC providing the wearable sensor with mechanical stability, leakage prevention, and antimicrobial properties. (**A**) Comparison of electromechanical characteristics of the PLMC with other reported elastic conductors. (**B**) Relative resistance changes of the PLMC subjected to cyclic stretching (200% maximum strain) for 2000 cycles. Inset: magnified resistance variations over a 10-cycle period. (**C**) Reliance test of the PLMC under harsh damage conditions, including puncturing with a scalpel, affects loading from a hammer strike, bending, and twisting. (**D**) Conceptual illustration of conventional nonporous (left) and our porous (right) LM composites upon stretching, showing that the porous structure provides damping effect to effectively reduce stresses on LM pathways. (**E**) Photographs of human skin after wearing nonporous (top) and porous (bottom) LM composites for 24 hours. The nonporous LM composites exhibit notable smearing effects, while our PLMC presents neglectable leakage. Scale bar, 5 mm. (**F**) Photographs of two adjacent conductive traces made of nonporous (top) and porous (bottom) LM composites before and after compression. Scale bars, 5 mm. (**G**) Viabilities of the gram-positive methicillin-resistant *S. aureus* (MRSA) and Gram-negative *P. aeruginosa* (PA) conditioned with various materials for 2 hours. *P < 0.05. (**H**) LIVE/DEAD staining of MRSA on PLMC with ε-polylysine (top) and control group (bottom) for 2 hours. Scale bar, 100 μm. Live and dead cells were stained green and red, respectively. (**I**) In vitro biocompatibility test using keratinocytes (HaCaT), monocytes (U937), and human dermal fibroblasts (HDF-α), cultured on various materials for 5 days. N.S. indicates no statistically significant differences. (**J**) LIVE/DEAD staining of HaCat cells on PLMC with ε-polylysin (top) and control (bottom). Scale bars, 100 μm. CFU, colony-forming units; CNTs, carbon nanotubes; NPs, nanoparticles.

Wearable sensors are subject to frequent mechanical deformations during exercise and daily activities, necessitating great deformation stability to maintain reliability and functionality. The employed PLMC (with fabrication process detailed in Materials and Methods) exhibits high conductivity (~2.0 × 10^5^ S/m) and stable electrical performance despite mechanical deformations, which benefits from the combined metallic and fluidic properties of LMs (*35*). The PLMC yields consistent electrical resistance under 300% strain (Fig. 2A), in stark contrast to other flexible conductive materials typically used in wearable devices, such as sputter-coated silver (Ag), silver nanoparticles (Ag NPs) (*36*), Ag flakes (*37*), and carbon nanotubes (CNTs) (*38*). These conventional materials show a notable resistance increase upon stretching. For instance, sputter-coated Ag demonstrates a 20-fold increase in resistance at a mere 14% strain, indicating a complete loss of functionality (Fig. 2A). Moreover, the PLMC demonstrates exceptional durability, with only a marginal resistance change of ~4.6% after 2000 stretching cycles at 200% strain (Fig. 2B). In addition, the PLMC exhibits a minimal electrical resistance change of less than 1% under harsh damage conditions, including puncturing with a scalpel, impact loading from a hammer strike, bending, and twisting (Fig. 2C). This remarkable stability can be attributed to the fluid-like nature of LMs, which reorients the tortuous conductive pathways under mechanical deformation, along with the energy-dissipating effect of the porous PU microstructures that dampens external mechanical impacts.

Besides maintaining stable resistance under mechanical deformations, our PLMC also provides leakage prevention ability, which is highly pursued by traditional LMs. Conventional LM composites are typically produced by blending micro- or nanoscale eutectic gallium-indium (EGaIn) particles with elastomers and then coalescing the particles to form percolation networks through mechanical or freezing sintering (fig. S3). Thus, mechanical deformations such as stretching and compression can damage the oxide shell of the LM, resulting in the LM being extruded along conductive pathways (left in Fig. 2D and fig. S4A). This undesired leakage not only leads to short circuits and corresponding malfunctions but also introduces contamination to skin and clothing, raising biosafety concerns. In contrast, our developed PLMC is synthesized using phase separation technology, which forms porous structures that provide damping effects to minimize leakage induced by mechanical deformations (right panel in Fig. 2D and fig. S4B). We compare the leakage prevention property of our PLMC with conventional nonporous LM composite (the LM content is identical to that in PLMC; see Materials and Methods for details). As shown in Fig. 2E, after wearing the materials on the skin for 24 hours, notable LM smearing is observed on the skin for the nonporous LM composite (top panel), while LM leakage is invisible for our PLMC (bottom). Moreover, the conventional nonporous LM composite exhibits notable leakage after compression, resulting in the short circuit of adjacent conductive traces (Fig. 2F, top). Conversely, compression has a negligible impact on the conductive traces of our PLMC (Fig. 2F, bottom). The excellent leakage prevention ability of the PLMC effectively mitigates the longstanding issue of LM composites, enhancing its applicability and feasibility in various wearable devices.

In addition to mechanical performance, antimicrobial properties are also crucial for on-skin sensors to ensure their prolonged and continuous usage, as the skin is constantly exposed to environments with pathogenic bacteria, posing the risk of infections. To mitigate this issue, we incorporate epsilon polylysine (ε-PL), a natural broad-spectrum antimicrobial peptide, into the PLMC to promote its antibacterial properties. The ε-PL–enhanced PLMC demonstrates remarkable antibacterial efficacy, achieving more than 99.9% bactericidal activity against typical skin pathogenic bacteria, including Methicillin-resistant *Staphylococcus aureus* (MRSA) and *Pseudomonas aeruginosa* (PA) (Fig. 2G). This antibacterial performance was visualized using a LIVE/DEAD fluorescence assay, revealing a notable decrease in MRSA cell count on the ε-PL–integrated PLMC compared to the control group (Fig. 2H). In addition, as ε-PL is considered safe as a food preservative, the incorporation of ε-PL has no adverse effect on the biocompatibility of the PLMC (*39*, *40*). Cytotoxicity assessments were performed using human keratinocyte (HaCaT), human dermal fibroblast (HDF-α), and human myeloid leukemia (U937) cell lines. The results indicate no significant differences (*P* > 0.05) in cell viability between cells cultured with the ε-PL–integrated PLMC and the control group (Fig. 2I). Moreover, fluorescence micrographs (Fig. 2J) show that HaCaT cells adhere well and spread uniformly on the ε-PL–integrated PLMC, with no visible signs of apoptosis or necrosis, thereby confirming outstanding biocompatibility.

### Wireless multiplexed sensing systems based on the high-order EP

**Fig. 3.**
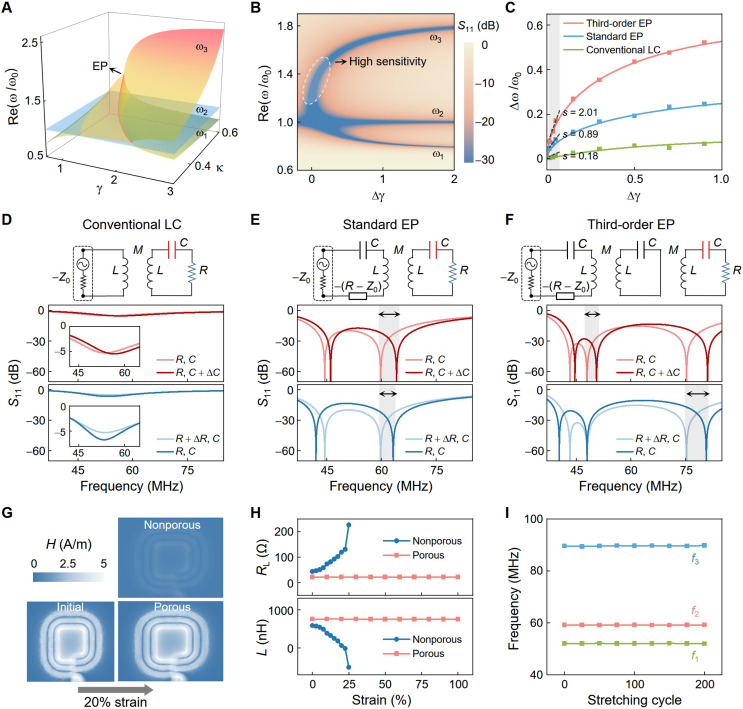
Mechanism and characterization of the wireless multiplexed sensing system enabled by the high-order EP. (**A**) Real parts of eigenfrequencies of the high-order EP-based system, where $\omega_{1}$ and $\omega_{3}$ bifurcate at the EP (pink line) and $\omega_{2}$ is locked at $\omega_{0}$ (blue surface). (**B**) Contours of the reflection coefficient (*S*_11_) of the high-order EP system, where eigenfrequencies appear of the minima of *S*_11_. Notably, $\omega_{3}$ is highly sensitive to variations in the gain-loss parameter γ ( $\Delta \gamma =\gamma -\gamma_{\mathrm{EP}}$ ). (**C**) Comparison of the primary resonance frequencies, i.e., $\omega_{3}$ of the high-order EP system, the second resonance frequency of the standard EP system, and the only resonance frequency of the conventional LC systems, as functions of perturbations in γ. The solid lines and dots represent theoretical and experimental results, respectively. In the region near the EP ( 0.015<Δγ<0.05 , gray-shaded), the third-order EP system demonstrates a sensitivity of 2.01 compared with 0.89 and 0.18 for the standard EP and LC systems under the same coupling coefficient (κ=0.5), giving 2.26-fold and 11.17-fold enhancements, respectively. (**D** to **F**) Comparison of sensing mechanisms of the conventional LC, standard, and high-order EP systems, showing that the high-order EP system can simultaneously respond to resistive and capacitive perturbations with high sensitivity, not possible with conventional LC or standard EP systems. (**G**) Magnetic field distributions of coil inductors, the main conductive component of the wearable sensor, made of nonporous and porous LM composites with the same LM content before and after 20% strain. (**H**) Resistance and inductance values of the coil inductor made of nonporous and porous LM composites as a function of uniaxial strains. The nonporous and porous LM composites have identical LM content. (**I**) Stability test of the high-order EP sensing system, demonstrating that its three resonance frequencies keep constant with stretching cycles.

Having introduced the wearability and biocompatibility of our sensor, we now discuss the systematic design, which uses the third-order EP topology to achieve highly sensitive and multiplexed sensing. As shown in Fig. 1C and fig. S1, the reader involves an active resonator (−*R*_1_, *L*_1_, and *C*_1_) and a neutral resonator (*L*_2_ and *C*_2_), while the sensor comprises one or multiple passive resonators (*xR*_3_, *xL*_3_, and *C*_3_/*x*). The positive number *x* serves as the scaling factor for enhanced design flexibility. The reader inductively couples to one sensing resonator at a time, forming a generalized third-order *PT-*symmetric (i.e., active-neutral-passive) system featuring the EP. The *PT* symmetry condition requires |−*R*_1_| = *xR*_3_ = *R*, *L*_1_ = *L*_2_ = *xL*_3_ = *L*, and *C*_1_ = *C*_2_ = *C*_3_/*x* = *C*. The negative resistance –*R*_1_ in the reader can be achieved by using a negative-resistance converter, or as in our design, using the negative resistance of a vector network analyzer (VNA) for simplified circuit design (|−*R*_1_| = *Z*_0_ = 50 ohm, where *Z*_0_ is the characteristic impedance of the VNA). Variations in the equivalent resistance (*R*_3_) and capacitance (*C*_3_) at a sensing resonator correspond to changes in biomarkers being monitored, such as skin temperature, Na^+^, K^+^, pH, NH_4_^+^, and glucose in sweat. These resistive and capacitive alterations can be simultaneously detected by tracking the shifts of multiple resonance frequencies in the reflection spectra measured at the reader. The resonance frequencies or eigenfrequencies of the third-order EP system can be derived from the system’s effective Hamiltonian (see note S1 for details), given by

$$\omega_{1}=\omega_{0}\sqrt{\frac{2\gamma^{2}-1-\sqrt{1-4\gamma^{2}+8\gamma^{4}\kappa^{2}}}{2\gamma^{2}\left(1-2\kappa^{2}\right)}}$$ (1A)

$$\omega_{2}=\omega_{0}$$ (1B)

$$\omega_{3}=\omega_{0}\sqrt{\frac{2\gamma^{2}-1+\sqrt{1-4\gamma^{2}+8\gamma^{4}\kappa^{2}}}{2\gamma^{2}\left(1-2\kappa^{2}\right)}}$$ (1C)

where $\omega_{0}=1/\sqrt{\text{LC}}$ is the angular resonance frequency of an LC resonator, $\gamma =R^{-1}\sqrt{L/C}$ represents the gain-loss parameter of the system, κ=M/L symbolizes the coupling strength between the adjacent resonators, and M is the mutual inductance. The eigenfrequencies $\omega_{1}$ and $\omega_{3}$ bifurcate at the EP [ $\gamma_{\text{EP}}={\left(2\kappa \right)}^{-1}\sqrt{1+\sqrt{1-2\kappa^{2}}}$ ] (pink line in Fig. 3A), particularly with $\omega_{3}$ changing rapidly with small variations in  γ, providing excellent sensitivity. Moreover, $\omega_{2}$ remains at $\omega_{0}$ (blue surface in Fig. 3A) that is independent of γ and κ values, offering the possibility for multiplexed sensing. At the region $\gamma >\gamma_{\text{EP}}$ , the three eigenfrequencies are purely real (Fig. 3A and fig. S5), manifesting as resonance frequencies that correlate to minimal reflection coefficients (*S*_11_) in the spectrum (Fig. 3B and note S2). It is therefore feasible to instantly obtain the eigenfrequencies of the system from the dips in the reflection spectrum.

We compare the primary resonance frequencies of the third-order EP system with those of the standard (second-order) EP and LC systems, demonstrating that with the identical coupling coefficient ( κ=0.5 ), the third-order EP system responds more sensitively to perturbations in γ (Fig. 3C and notes S2 and S3). When operating in the vicinity of the EP (gray-shaded region, Fig. 3C), the measured sensitivity (*s*), defined as the slope of frequency versus Δγ , is 2.01 for the third-order EP system, compared with 0.89 and 0.18 for the standard EP and LC systems, respectively. These values correspond to 2.26-fold and 11.17-fold sensitivity enhancements (fig. S6). The sensitivity of the high-order EP system could even be improved by applying larger κ or putting the coil inductors closer (fig. S7). The advantages of the proposed high-order EP-based sensing system can be further noticed from Fig. 3 (D to F). We use the same coupling strength; scaling factor; initial *R*, *L*, and *C* values; and perturbations (Δ*R* and Δ*C*). It turns out that by tracking the resonance frequency of the LC sensing system, one can only monitor the capacitive perturbation (Δ*C*) with low sensitivity. The resistance perturbation (Δ*R*) has a neglectable impact on the resonance frequency of the LC system, only slightly varying the modulation depth of *S*_11_. In contrast, the standard EP system, which has a pair of resonance frequencies related to both *R* and *C* values (note S3), enables the detection of resistive or capacitive perturbations with enhanced sensitivity. However, either Δ*R* or Δ*C* can cause shifts of the two resonance frequencies, making the standard EP system not capable of distinguishing or simultaneously monitoring resistive and capacitive perturbations. Notably for our high-order EP system, given the fixed inductance, the second resonance frequency $\omega_{2}$ is solely related to *C*, and thus, Δ*C* can be monitored by tracking the shift of $\omega_{2}.$ Once the capacitance is obtained, the resistance value *R* can be simply obtained from $\omega_{3}.$ In this way, the high-order EP system allows sensitive monitoring of both resistance and capacitive variations with a single “RLC” sensing resonator (fig. S8). In addition, thanks to the fully passive circuit architecture, the system exhibits minimal noise and frequency fluctuations, ensuring excellent stability even when operating in close proximity to the EP (fig. S9 and note S4).

To further validate its potential on skin-interfaced monitoring, we evaluate the performance of the wearable sensor under strain, a mechanical deformation that commonly occurs during wear. Our investigations focus on 220- and 770-nH coil inductors, the primary conductive components of the wearable sensor. Maxwell simulation reveals that the coils made of PLMC exhibit minimal alteration in magnetic field distribution under 20% strain, while their nonporous counterparts (with identical LM content) show a notable reduction in magnetic field intensity (Fig. 3G and fig. S10). This phenomenon can be attributed to the resistance (*R*_L_) and inductance (*L*) behaviors of the coil inductors. Under 100% strain, the porous coil maintains stable *R*_L_ and *L* values, while the nonporous one exhibits notable changes in *R*_L_ and *L*, ultimately losing functionality at 20% strain (Fig. 3H and fig. S11). Moreover, the PLMC endows the coil with exceptional durability during repeated stretching and bending tests (figs. S12 and S13). The wearable sensor accordingly demonstrates reliable and durable performance under strain, with the three resonance frequencies ( $f_{n}=\omega_{n}/2\pi ,n=1,2,3$ ) remaining nearly unchanged while enduring 200 stretching cycles (100% maximum strain) (Fig. 3I). The exceptional stretchability—combined with the soft, nonleakage, and antibacterial properties of the PLMC—renders the sensor highly suitable for prolonged skin-interfaced monitoring and other wearable applications.

### Wireless perspiration monitoring during physical exercise

**Fig. 4.**
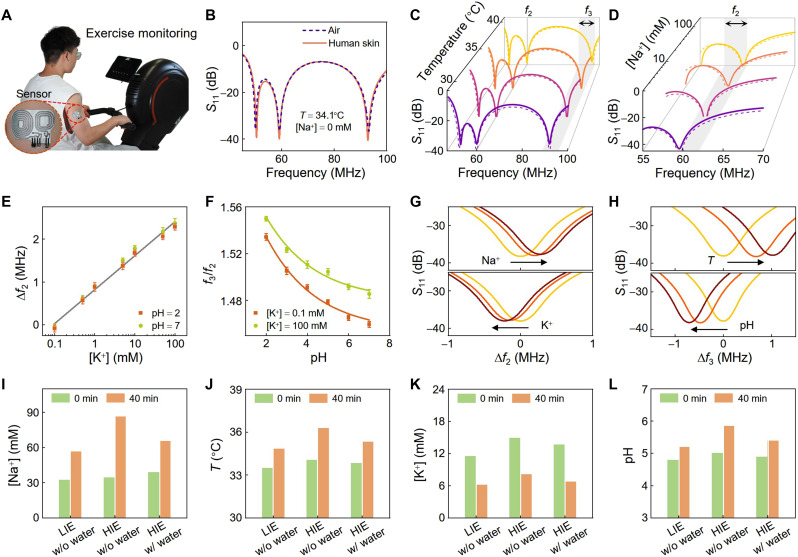
Monitoring of skin temperature, sweat [Na^+^], [K^+^], and pH during exercise. (**A**) Photograph of a volunteer wearing the soft multiplexed sensor during exercise. (**B**) Reflection spectra of the proposed wireless sensing system when the sensor is placed in the air and attached to the human skin. The resonance frequencies are unaffected by the presence of human body. (**C**) Reflection coefficients (*S*_11_) with various temperature conditions, showing that the third resonance frequency $f_{3}$ notably shifts with temperature, while the second resonance frequency $f_{2}$ remains constant. (**D**) *S*_11_ with various Na^+^ concentrations, showing that $f_{2}$ can respond to variations in [Na^+^]. (**E**) Shift of the second resonance frequency ( $\Delta f_{2}$ ) with respect to K^+^ concentration, which remain consistent with different pH conditions. (**F**) Frequency ratio $f_{3}/f_{2}$ as a function of pH with different [K^+^]. Once [K^+^] is determined by $f_{2}$ (E), the relationship between $f_{3}/f_{2}$ and pH is transparent. (**G** and **H**) Resonance frequency shifts during 40-minite low-intensity exercise (LIE), indicating changes in skin temperature (*T*), sweat [Na^+^], [K^+^], and pH. (**I** to **L**) Sweat [Na^+^], skin temperature, sweat [K^+^], and pH during 40-min LIE, high-intensity exercise (HIE) without (w/o) water intake, and HIE with (w/) water intake. The monitoring takes 20-min time interval, with detailed results provided in fig. S27.

The efficacy of our proposed sensing system is primarily validated by perspiration monitoring, as sweat is a bodily fluid rich in essential electrolytes (such as Na^+^ and K^+^) and metabolites (such as glucose) (*11*, *12*, *41*–*44*). Monitoring these biomarkers offers valuable physiological insights for personalized health management (*45*–*48*), disease prevention and diagnosis (*49*–*51*), and drug abuse detection (*52*–*54*). Here, we use the proposed system to monitor skin temperature (*T*) and sweat biomarkers (Na^+^, K^+^, and pH) during physical exercise (Fig. 4A). As previously discussed, these four biomarkers can be monitored by sequentially interrogating two sensing resonators with the same reader: one comprising temperature and Na^+^ transducers and the other including pH and K^+^ transducers. The temperature and pH variations are directly reflected in the resistance of a negative temperature coefficient thermistor and a custom-made pH-sensitive resistor, respectively (figs. S1 and S14). Fluctuations in Na^+^ and K^+^ concentrations induce changes in the electrical potential difference between electrodes, serving as the bias voltage of varactors (fig. S15). Thus, the Na^+^ and K^+^ concentrations are correlated with the capacitance of the varactors (figs. S1 and S16). The resistances between the Na^+^ and K^+^ electrodes are consistently at megohm levels and have negligible influence on overall system performance (fig. S17). Considering the equivalent resistance and capacitance values of the transducers and coils, the *T*-Na^+^ and pH-K^+^ sensing resonators attain γ values of 1.83 at *T* = 25°C and [Na^+^] = 2 mM and 1.72 at pH = 7 and [K^+^] = 0.1 mM, respectively. These resistance and capacitance values are designed to ensure our sensing system operates close to the EP (i.e., $\gamma \to \gamma_{\text{EP}}$ ) for optimal sensitivity and accurate biomarker monitoring. The coupling coefficient κ is fixed at 0.45 for biomarker monitoring, corresponding to a ~3-mm center-to-center distance between reader and sensor coils. This alignment is practically ensured by using spacers and markers on the reader, which reproduces the relative coil position while preserving comfort and wearability (fig. S18). We note that the system can also operate at smaller coupling coefficients (Fig. 3A), which extend the readout distance. For instance, κ=0.2 corresponds to a distance of ~8 mm (fig. S19A). While a reduction in κ moderately decreases the maximum achievable sensitivity, the system maintains its capability for multiparameter monitoring and provides improved sensitivity compared with the standard EP and LC systems at the same κ . Furthermore, the coupling coefficients between the reader and nontargeted sensor and between the two sensors are consistently around or below 0.02 (fig. S19B), indicating negligible mutual interference. This is also supported by the following experimental observations, in which the measured resonance frequencies agree well with theoretical predictions and shift accurately with parameter variations.

Primarily, we demonstrate that the proposed sensing system exhibits consistent resonance frequencies when the sensor is positioned in the air or in contact with human skin, suggesting minimal influence of the human body on monitoring results (Fig. 4B). This reliability stems from the inductive coupling between the reader and sensor, wherein the coupling strength is impervious to the dielectric properties of human tissues. Moreover, thanks to the multifrequency resonance behavior (Eqs. 1A to 1C), the system is capable of monitoring two parameters simultaneously with negligible cross-talk. Specifically, the third resonance frequency $f_{3}$ is highly responsive to temperature variations, whereas the second resonance frequency $f_{2}$ remains unaffected by temperature and responds solely to Na^+^ concentration (Fig. 4, C and D, and figs. S20 to S23). Similarly, the relationship between $f_{2}$ and K^+^ concentration is independent of pH alterations (Fig. 4E and fig. S24). Upon determining K^+^ concentration, the correlation between $f_{3}/f_{2}$ and pH becomes apparent, enabling the determination of pH levels via tracking $f_{3}/f_{2}$ (Fig. 4F and fig. S25). The results show the capacity of the high-order EP system in monitoring temperature, Na^+^, pH, and K^+^ with a compact dual-resonator sensor. The sensitivities for temperature, Na^+^, pH, and K^+^ monitoring are 0.39 MHz/°C at [Na^+^] = 2 mM, 1.13 MHz per decade, 0.88 MHz/pH at [K^+^] = 0.1 mM, and 0.79 MHz per decade, respectively. The high-order EP-based sensing system exhibits particularly high sensitivity for temperature and pH (i.e., resistive) detection, owing to the eigenfrequency bifurcation effect at the EP, markedly surpassing that of a traditional LC sensing system (*5*, *18*). By approaching the divergent point, i.e., $\kappa \to \sqrt{2}/2$ in (Eqs. 1A and 1B), the eigenfrequency bifurcation can be further boosted, corresponding to enhanced sensitivity (*55*, *56*). In addition, the system exhibits sufficiently high resolutions, quality (*Q*) factors, and signal-to-noise ratios (SNRs), enabling the detection of subtle biomarker variations (fig. S26)

Further, the ability of our system is validated for in situ perspiration monitoring during exercise. The sensor conforms to human skin, with its sensing region contacting accumulated sweat during exercise. A PU film encapsulates this region to prevent sweat evaporation and protect it against environmental contaminants (Fig. 4A). During a 40-min low-intensity exercise (LIE), we observe clear frequency shifts induced by variations in skin temperature, sweat [Na^+^], [K^+^], and pH. Specifically, for the *T*-Na^+^ sensing resonator, both the second and third resonance frequencies exhibit upward shifts over time, indicating reductions in equivalent capacitance *C*_3_ and resistance *R*_3_ values, respectively (top panels in Fig. 4, G and H). These results correlate with increases in temperature and Na^+^ concentrations, as revealed by the established relationship between *C*_3_ and *R*_3_ values and biomarkers (figs. S14 and S16). On the other hand, for the pH-K^+^ sensing resonator, both $f_{2}$ and $f_{3}$ demonstrate downward shifts during the exercise, indicating increases in *C*_3_ and *R*_3_, associated with the decrease in K^+^ concentration and the increase in pH (lower panels in Fig. 4, G and H). These trends are more visible from the left columns in Fig. 4 (I to L), and gray lines in fig. S27. Following a 40-min LIE, the skin temperature rises from around 33.5° to 34.8°C. Meanwhile, there exhibits an increase in sweat [Na^+^] from 32.3 to 56.4 mM, and a decrease in [K^+^] from 11.5 to 6.2 mM, a result of electrolyte balance adjustment. These observations align with the commonly acknowledged change trends (*11*). In addition, the sweat pH remains relatively stable, exhibiting only a marginal rise from 4.8 to 5.2, owing to the accumulation of alkaline metabolites such as NH_4_^+^. We further compare these biomarker variations to those recorded during high-intensity exercise (HIE; middle columns in Fig. 4, I to L, red lines in figs. S27 and S28). The results reveal similar alteration trends of biomarkers during low- and high-intensity exercises, while the HIE brings more notable variations that may be associated with increased sweat rate. Following a 40-min HIE, the sweat [Na^+^] rises nearly 2.5 times the baseline, accompanied by clear variations in skin temperature, sweat [K^+^], and pH. This may be a result of increased Na^+^ concentration in blood serum and an indication of dehydration. Regular water intake (120 ml per 5 min) can mitigate the dehydration, leading to the dilution effect that stabilizes temperature, Na^+^, K^+^, and pH as exercise persists (right columns in Fig. 4, I to L, blue lines in figs. S27 and S29). These measurement results are comparable to those obtained from commercial instruments, confirming the reliability of our sensing system (fig. S30).

### Long-term sweat glucose and ammonium monitoring

**Fig. 5.**
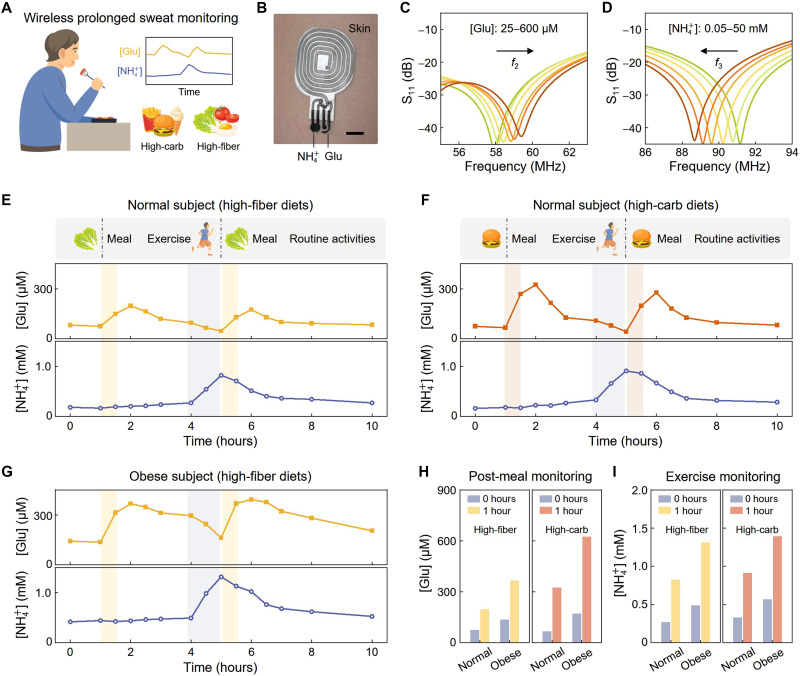
Prolonged monitoring of sweat glucose and [NH_4_^+^] for normal and obese subjects during daily activities. (**A**) Schematic illustration of the wireless system for monitoring sweat glucose and [NH_4_^+^] of a subject with high-fiber and carbohydrate (high-carb) dietary intakes. (**B**) Photogragh of the wearable sweat sensor, using a single sensing coil for simultaneous monitoring of sweat glucose and [NH_4_^+^]. Scale bar, 1 cm. (**C** and **D**) Reflection spectra of the wireless sensing system, showing that the second and third resonance frequencies can notably shift with variations in [Glu] and [NH_4_^+^], respectively. In (C), [NH_4_^+^] = 50 mM, and in (D), [Glu] = 600 μM. (**E** and **F**) Dynamic changes in sweat [Glu] and [NH_4_^+^] during daily activities, including food intakes, exercises, and other routine activities (i.e., working, talking, etc.). The monitoring was performed on a normal subject for 2 days with different dietary intakes; one with a high-fiber diet (E) and the other with a high-carbohydrate diet (F). (**G**) Analysis of sweat [Glu] and [NH_4_^+^] for an obese subject with high-fiber dietary intakes, indicating relatively higher glucose levels compared to that of the normal subject. (**H** and **I**) Sweat [Glu] and [NH_4_^+^] of the normal and obese subjects post the first high-fiber and high-carbohydrate meals (H) and during 1-hour physical exercise (I).

To further demonstrate the ability of our system in prolonged and continuous monitoring, we use it for monitoring sweat glucose and NH_4_^+^ variations over 10-hour time periods and compare these variations in normal and obese subjects with different dietary intakes (Fig. 5A). In line with previous designs, the translation of glucose levels is achieved through a pair of glucose electrodes serving as the bias of a varactor and the NH_4_^+^ concentration is assessed using a custom-made NH_4_^+^-sensitive resistor (fig. S1). Owing to the high-order EP mechanism, our wireless sensing system allows simultaneous monitoring of glucose and NH_4_^+^ levels with a single sensing resonator (Fig. 5B and fig. S1), whose equivalent capacitance and resistance are proportional to glucose and NH_4_^+^ concentrations, respectively (fig. S31). As observed in the measured reflection spectra, the second and third resonance frequencies shift sensitively with the glucose and NH_4_^+^ variations, respectively, consistent with our theoretical predictions (Fig. 5, C and D, and figs. S32 and S33). Furthermore, the wearable sensor—with softness, stretchability, leakage prevention, and antibacterial properties—is highly comfortable and biocompatible, well-suited for extended wear and continuous monitoring applications.

Our wireless sensing system was used to monitor sweat glucose and NH_4_^+^ levels in a normal 28-year-old subject over 2 days, during which the subject consumed distinct diets. On the first day, the subject ingested a high-fiber diet including vegetables and salads, while on the second day, the subject consumed high-carbohydrate foods (e.g., hamburgers, fries, and noodles). Following both high-fiber and high-carbohydrate meals, there exhibits an immediate increase in glucose levels, which subsequently decline to near-baseline levels within 1 to 2 hours postingestion (Fig. 5, E and F, and fig. S34). This pattern suggests typical digestive and insulin responses. However, it is noteworthy that high-carbohydrate meals induce a more notable rise in glucose, with peak concentrations reaching ~300 μM, in contrast to high-fiber meals leading to peak glucose levels below 200 μM. As expected, physical exercise leads to a rapid reduction in glucose levels and a concurrent rise in sweat [NH_4_^+^] (Fig. 5, E and F, and fig. S35), reflecting promoted metabolic activity and blood glucose consumption during exercise. For routine activities such as reading, working, and conversation, the glucose and NH_4_^+^ levels are relatively stable with only minor variations.

Further, we examined glucose and NH_4_^+^ levels in a 45-year-old obese subject. In comparison to the normal subject, the obese individual exhibits a more rapid increase in glucose levels postprandially (Fig. 5, G and H, and fig. S34). Moreover, the sweat glucose of the obese subject remains relatively high 2 hours postmeal, which may indicate early symptoms of hyperglycemia or diabetes (Fig. 5G and fig. S34). High-carbohydrate diets may exacerbate this situation, leading to a high glucose level of ~600 μM (Fig. 5H). In addition, the sweat [NH_4_^+^] of the obese subject is relatively higher than that of the normal subject, with moderate influence from dietary intake (Fig. 5, G and I, and fig. S35). In general, the proposed wireless sensing system demonstrates robust capabilities for extended monitoring of sweat glucose and [NH_4_^+^] during routine activities, offering valuable insights into physical health. Our wireless, sensitive, and multiplexed sensing system is highly transformative, with potential applications beyond monitoring skin temperature, Na^+^, K^+^, pH, NH_4_^+^, and glucose. By modulating sensing electrodes and transducers, the system could monitor a broader spectrum of biomarkers, such as uric acid and lactose levels, expanding its applicability in various wearable and biomedical contexts.

## DISCUSSION

Despite the growing demand for wearable and on-skin wireless sensors, their utility remains constrained by compromised sensing functionality and wearability. In this work, we have developed a wearable, wireless, battery-free sensing system, taking advantage of the high-order EP singularity in non-Hermitian domain to achieve highly sensitive and multiplexed sensing performance while using the soft, stretchable PLMC and porous PU to ensure mechanical stability, wearing comfort, and biocompatibility. We have revealed that the existence of the high-order EP enhances sensitivity by an order of magnitude relative to traditional LC sensing systems. Concurrently, the three distinct resonance frequencies of the high-order EP system enable simultaneous detection of resistive and capacitive parameters within a single resonator, reducing sensor size by half compared to existing resonator-based sensing systems. In addition, the soft, stretchable features of PLMC endow the wearable sensor with superior wearing comfort and robustness against mechanical deformations (e.g., stretching, bending, and pressing). The porous structures of the PLMC afford the sensor high leakage resistance, while the incorporation of ε-PL confers antimicrobial and biocompatible properties. These combined advantages make our system highly desirable for skin-interfaced wireless sensing applications. We have implemented our system to wirelessly monitor skin temperature and sweat electrolytes (e.g., Na^+^, K^+^, and pH) during 40-min physical exercises and sweat glucose and NH_4_^+^ over 10-hour daily activities. The system exhibits high sensitivity and accurate discrimination of these biomarkers through a simple and compact sensor design, providing valuable information for health assessment. Furthermore, the multifaceted sensing capability may extend beyond perspiration monitoring. By modulating the sensing electrodes, this wireless sensing platform could be adapted to monitor other biomarkers, finding applications in various wearable and implantable biomedical sensing scenarios. A possible future direction is the realization of a portable standalone reader that integrates a compact RF source, directional coupler, reflection detector, and a microcontroller-driven frequency tracking algorithm. This would eliminate reliance on a laboratory-grade VNA and enable low-cost, portable sensing in clinical settings.

## MATERIALS AND METHODS

### Materials

Bis(2-ethylehexyl) sebacate (DOS), sodium tetrakis [3,5-bis(trifluoromethyl)phenyl] borate (Na-TFPB), polyvinyl chloride (PVC), sodium tetraphenylborate (NaTPB), cyclohexanone, polyvinyl butyral resin BUTVAR B-98 (PVB), ammonium peroxydisulfate, iron (III) *p*-toluenesulfonate hexahydrate, aniline, NaCl, and KCl were purchased from Sigma-Aldrich. Polyaniline base, acetic acid, dimethyl sulfoxide, tetrahydrofuran, and cyclohexanone phosphate-buffered saline (PBS) solution were obtained from Thermo Fisher Scientific. Glucose oxidase was obtained from Toyobo Corp.

### Fabrication of the PLMC and conductive traces

The PLMC was prepared by drop casting a mixture of PU [70 mg ml^−1^ in tetrahydrofuran (THF)] and EGaIn (75.5% Ga, 24.5% In) solution (200 mg ml^−1^ in 1-butanol, dispersed by tip sonication) with a volume ratio of 5:4 onto aluminum foil, followed by drying at room temperature. For the ε-PL–incorporated PLMC, ε-PL (7 mg ml^−1^) was added to the PU solution before the mixture. As a comparison, we also fabricated non-PLMCs using the same procedure as for PLMC, except that 1-butanol was replaced with THF. For the fabrication of coils and conductive traces, a CO_2_ laser was used to cut and define the obtained PLMC to prepare the coil and conductive traces for the sensor. The defined coil and conductive traces were then transferred to the porous PU substrates using waterborne PU as an adhesive.

### Fabrication of the biochemical transducers

The Na^+^, K^+^, and glucose transducers were prepared using previously reported methods. Briefly, the PU-transferred laser-induced graphene (LIG) electrodes served as the base electrodes, with specific modifications applied for each type of transducer. For the Na^+^ transducer, a Na^+^ selective membrane cocktail (1 mg of Na ionophore, 0.55 mg of Na-TFPB, 33 mg of PVC, and 65.45 mg of DOS in 660 μl of THF) was added to the working electrodes, while a K^+^-selective membrane precursor solution (2 mg of valinomycin, 0.5 mg of NATPB, 32.7 mg of PVC, and 64.7 mg of DOS in 350 μl of cyclohexanone) was applied to the working electrodes of the K^+^ sensors. To prepare glucose transducers, gold nanoparticles (25 nm) were first sputter-coated on the LIG electrodes, and 5 μl of cocktail (10 mg of Prussian blue and 5 mg of chitosan dissolved in 1 ml of 0.1 M acetic acid) was then drop-casted. Next, 5 μl of glucose oxidase solution was added and dried at 4°C overnight, followed by the addition of 3 μl of 0.5% Nafion solution. Here, the glucose oxidase solution was prepared by mixing glucose oxidase (10 mg ml^−1^ in PBS) with chitosan/CNT solution [1% chitosan and multiwalled CNT (5 mg ml^−1^) in 0.1 M acetic acid] at a volume ratio of 1:1. The resistance-type NH_4_^+^ and pH transducers were prepared by drop-casting the corresponding cocktail solutions onto the transferred LIG interdigital electrodes, followed by washing and air drying. The cocktail for the pH transducer was prepared by dispersing polyaniline emeraldine base (PANI-ES, 10 mg ml^−1^) and conductive carbon black (Super-P, 10 mg ml^−1^) into a 0.1 M HCl solution. The cocktail for the NH_4_^+^ transducer was prepared by dispersing *p*-toluene sulfonate hexahydrate–doped polyaniline (PTS-PANI) into deionized water at a concentration of 20 mg ml^−1^.

### Antibacterial activity

The antibacterial effectiveness of PLMC was investigated using two bacterial strains: MRSA (USA300) and *Pseudominas aeruginosa* E411-17 (PA). Each sample was separately added to 1 ml of the respective bacterial suspension (10^8^ CFU/ml) in PBS and incubated at 37°C for 6 hours. After incubation, the bacterial solution was diluted 1000, and 10 μl of the diluted solution was spread onto an LB agar plate.

### Cytocompatibility test

The cytotoxicity of the developed materials was assessed in three different cell lines: HaCaT, HDF, and U937, as reported (*57*). Briefly, HaCaT and HDF cells were cultured in Dulbecco’s modified Eagle’s medium supplemented with 10% fetal bovine serum (FBS) and 1% penicillin-streptomycin, while U937 cells were grown in RPMI 1640 medium supplemented with 10% FBS and 1% penicillin-streptomycin. The cells were seeded in a 48-well plate at a density of 10^4^ cells per well and sterilized, and precut (1 cm by 1 cm) samples were transferred to each well using well-inserts. The cells were incubated at 37°C for 3 days, and cell toxicity was determined by adding 20 μl of Cell Counting Kit-8 (CCK-8) per well, with absorbance measured at 450 nm using a microplate reader. The obtained data were evaluated for statistical significance using a two-way analysis of variance (ANOVA), followed by Tukey’s post hoc multiple comparison test, with differences considered significant at *P* < 0.05. In addition, following staining with the LIVE/DEAD cell viability kit, the cells were examined using an inverted fluorescence microscope.

### System design and numerical simulations

The systematic schematic of the perspiration monitoring system is shown in fig. S1. The inductors in the sweat sensor use rectangular coil designs with arc-shaped corners to save space and ensure optimal performance. Specifically, two distinct coil designs are used; one with three turns, 10-mm inner length, 2-mm line width, 0.5-mm spacing, 1.5-mm inner arc radius for the detection of skin temperature and sweat Na^+^, and the other with six turns, 6-mm inner length, 2-mm line width, 0.5-mm spacing, 0.5-mm inner arc radius for the detection of pH and K^+^, NH_4_^+^, and glucose. The two coils with are measured 220 and 770 nH, respectively, with resistance induced by the conductive PLMC. A negative temperature coefficient thermistor (NTCG103EH300JT1, TDK Corporation) with a resistance of 30 ohms at 25°C is used for temperature detection. Macom varactor diodes are connected to the biochemical transducers, specifically MAVR-000405-0287FT for Na^+^ and MAVR-045441-0287FT for K^+^ and glucose, for converting the electrical potential between the electrodes into capacitance. The magnetic field distributions of the inductive coils are simulated in Ansys Maxwell. The simulations of the reflection coefficients of the sweat sensing system are performed in Advanced Design System. The theoretical calculations of the reflection coefficients and eigenfrequencies of the sweat sensing system are performed in Mathematica.

### Characterizations and measurements

Mechanical properties were characterized using a Mark-10 ESM303 tensile tester. Electrical conductance was measured by a digital source meter (2604B, Keithley Instruments). The impedance of the coil and the reflection coefficients of the sweat sensing system were measured using a VNA (N5242B PNA-X Network Analyzer, Keysight). The on-body performance of the sweat sensing system was measured using a portable VNA (LibreVNA). Both VNAs used in this study provide a stable 50-ohm port and sufficient frequency resolution in the operation range, ensuring that the extracted resonance frequency and third-order EP behavior are unaffected by the specific instrument used. Resonance frequencies were determined using a LabVIEW algorithm, which identifies the raw minimum from an initial sweep of 2000 points, applies a second-degree polynomial fit over a 3-dB range around the minimum (1000 points), and takes the minimum of the fitted curve as the resonance dip. The total time required to determine a resonance frequency, including the initial sweep and peak detection, is ~500 ms.

### Experiments on human subjects

The on-body evaluations of skin-interfaced devices on human participants were conducted under approval from Institutional Review Board at the University of Illinois Chicago (STUDY2025-0088). All human subjects gave written and informed consent before participation in the studies.

## Supplementary Materials

### This PDF file includes:

Supplementary Notes S1 to S4 Figs. S1 to S35 Tables S1 and S2 References
