# Supplementary material for: A highly sensitive and multiplexed wireless sensing system with skin-like compliance and stretchability for wearable applications
Source: Sci Adv. 2025 Oct 29;11(44):eadt4923. doi: 10.1126/sciadv.adt4923 (PMC12571053; doi:10.1126/sciadv.adt4923)
Supplement: Supplementary file 1 — Supplementary Notes S1 to S4 Figs. S1 to S35 Tables S1 and S2 References [file sciadv.adt4923_sm.pdf]

Science Advances
AAAS

Supplementary Materials for

A highly sensitive and multiplexed wireless sensing system with skin-like compliance and stretchability for wearable applications

Zhilu Ye *et al.*

Corresponding author: Pai-Yen Chen, pychen@uic.edu; Zheng Yan, yanzheng@missouri.edu;
Wei Gao, weigao@caltech.edu

Sci. Adv. **11**, eadt4923 (2025)
DOI: 10.1126/sciadv.adt4923

This PDF file includes:

Supplementary Notes S1 to S4
Figs. S1 to S35
Tables S1 and S2
References

Note S1. Theoretical calculation of the eigenfrequencies of the generalized third-order EP-based monitoring system

The generalized third-order EP-based monitoring system in Fig. 1C consists of active "–RLC" (gain), "LC" (neutral), and passive "RLC" (loss) resonators which are inductively coupled with each other. The system can be described by Kirchhoff's laws, as follows:

$$\begin{aligned}
\frac{d^2q_1}{d\tau^2} &= -\frac{1-\kappa^2}{1-2\kappa^2}q_1 + \frac{\kappa}{1-2\kappa^2}q_2 - \sqrt{x}\frac{\kappa^2}{1-2\kappa^2}q_3 + \frac{1-\kappa^2}{\gamma(1-2\kappa^2)}\dot{q}_1 - \sqrt{x}\frac{\kappa^2}{\gamma(1-2\kappa^2)}\dot{q}_3,\\
\frac{d^2q_2}{d\tau^2} &= \frac{\kappa}{1-2\kappa^2}q_1 - \frac{1}{1-2\kappa^2}q_2 + \sqrt{x}\frac{\kappa}{1-2\kappa^2}q_3 - \frac{\kappa}{\gamma(1-2\kappa^2)}\dot{q}_1 + \sqrt{x}\frac{\kappa}{\gamma(1-2\kappa^2)}\dot{q}_3,\\
\frac{d^2q_3}{d\tau^2} &= -\frac{1}{\sqrt{x}}\frac{\kappa^2}{1-2\kappa^2}q_1 + \frac{1}{\sqrt{x}}\frac{\kappa}{1-2\kappa^2}q_2 - \frac{1-\kappa^2}{1-2\kappa^2}q_3 + \frac{1}{\sqrt{x}}\frac{\kappa^2}{\gamma(1-2\kappa^2)}\dot{q}_1 - \frac{1-\kappa^2}{\gamma(1-2\kappa^2)}\dot{q}_3,
\end{aligned} \tag{S1}$$

where q_1, q_2, and q_3 correspond to the charge stored on the capacitors in the "–RLC", "LC", and "RLC" resonators, $\tau \equiv \omega_0 t$, ω_0 $(=1/\sqrt{LC})$ is the resonant angular frequency of the "LC" oscillator, γ $(=R^{-1}\sqrt{L/C})$ is the effective Q-factor or gain-loss parameter of the resonant tank, κ $(=M/L)$ is the coupling strength between the adjacent resonators, M is the mutual inductance, and x is the scaling factor. On condition that x = 1, the system converges to parity-time (PT)-symmetric configuration, invariant under a combined parity $\mathcal{P}$ (i.e., $q_1 \leftrightarrow q_3$) and time reversal $\mathcal{T}$ (i.e., $t \to -t$) transformation. We consider this as a special case of the generalized PT symmetry, where Eq. (S1) can be casted into the Liouvillian formalism, given by:

$$\mathcal{L}\boldsymbol{\Psi} = \frac{d\boldsymbol{\Psi}}{d\tau},$$

$$\mathcal{L} = \begin{pmatrix}
0 & 0 & 0 & 1 & 0 & 0\\
0 & 0 & 0 & 0 & 1 & 0\\
0 & 0 & 0 & 0 & 0 & 1\\
-\frac{1-\kappa^2}{1-2\kappa^2} & \frac{\kappa}{1-2\kappa^2} & -\frac{\kappa^2}{1-2\kappa^2} & \frac{1-\kappa^2}{\gamma(1-2\kappa^2)} & 0 & -\frac{\kappa^2}{\gamma(1-2\kappa^2)}\\
\frac{\kappa}{1-2\kappa^2} & -\frac{1}{1-2\kappa^2} & \frac{\kappa}{1-2\kappa^2} & -\frac{\kappa}{\gamma(1-2\kappa^2)} & 0 & \frac{\kappa}{\gamma(1-2\kappa^2)}\\
-\frac{\kappa^2}{1-2\kappa^2} & \frac{\kappa}{1-2\kappa^2} & -\frac{1-\kappa^2}{1-2\kappa^2} & \frac{\kappa^2}{\gamma(1-2\kappa^2)} & 0 & -\frac{1-\kappa^2}{\gamma(1-2\kappa^2)}
\end{pmatrix}, \tag{S2}$$

where $\boldsymbol{\Psi} \equiv (q_1, q_2, q_3, \dot{q}_1, \dot{q}_2, \dot{q}_3)^T$. Moreover, an effective Hamiltonian can be defined as: $H_{\text{eff}} = i\mathcal{L}$, which has a non-Hermitian form $H_{\text{eff}}{}^{\dagger} \neq H_{\text{eff}}$. The effective Hamiltonian is symmetric with respect to the combined $\mathcal{PT}$ transformation, i.e., $[\mathcal{PT}, H_{\text{eff}}] = 0$, with

$$\mathcal{P} = \begin{pmatrix} \mathbf{J} & 0\\ 0 & \mathbf{J}\end{pmatrix} \text{ and } \mathcal{T} = \begin{pmatrix} \mathbf{I} & 0\\ 0 & -\mathbf{I}\end{pmatrix}\mathcal{K}, \tag{S3}$$

where $\mathbf{J}$ is the 3×3 anti-diagonal matrix with unit entries, $\mathbf{I}$ is the 3×3 identity matrix, and $\mathcal{K}$ performs the operation of complex conjugation.

Additionally, when x is an arbitrary positive real number unequal to one, the system in a

generalized form, or *PT*-reciprocal scaling (*PTX*) symmetry, invariant under a combined parity $\mathcal{P}$ (i.e., $q_1 \leftrightarrow q_3$), time reversal $\mathcal{T}$ (i.e., $t \rightarrow -t$) and reciprocal-scaling $\mathcal{X}$ (i.e., $q_1 \rightarrow x^{-1/2} q_1$, $q_2 \rightarrow x^{-1/2} q_2$, $q_3 \rightarrow x^{1/2} q_3$) transformation. In this case, the "RLC" resonator is scaled following a specific rule, i.e., $R \rightarrow xR$, $L \rightarrow xL$, $C \rightarrow x^{-1}C$, with x serving as the reciprocal scaling factor. Similarly, Eq. (S1) can be casted into the Liouvillian formalism, given by:

$$\mathcal{L}'\mathbf{\Psi}' = \frac{d\mathbf{\Psi}'}{d\tau},$$

$$\mathcal{L}' = \begin{pmatrix} 0 & 0 & 0 & 1 & 0 & 0 \\ 0 & 0 & 0 & 0 & 1 & 0 \\ 0 & 0 & 0 & 0 & 0 & 1 \\ -\frac{1-\kappa^2}{1-2\kappa^2} & \frac{\kappa}{1-2\kappa^2} & -\sqrt{x}\frac{\kappa^2}{1-2\kappa^2} & \frac{1-\kappa^2}{\gamma(1-2\kappa^2)} & 0 & -\sqrt{x}\frac{\kappa^2}{\gamma(1-2\kappa^2)} \\ \frac{\kappa}{1-2\kappa^2} & -\frac{1}{1-2\kappa^2} & \sqrt{x}\frac{\kappa}{1-2\kappa^2} & -\frac{\kappa}{\gamma(1-2\kappa^2)} & 0 & \sqrt{x}\frac{\kappa}{\gamma(1-2\kappa^2)} \\ -\frac{1}{\sqrt{x}}\frac{\kappa^2}{1-2\kappa^2} & \frac{1}{\sqrt{x}}\frac{\kappa}{1-2\kappa^2} & -\frac{1-\kappa^2}{1-2\kappa^2} & \frac{1}{\sqrt{x}}\frac{\kappa^2}{\gamma(1-2\kappa^2)} & 0 & -\frac{1-\kappa^2}{\gamma(1-2\kappa^2)} \end{pmatrix}. \quad \text{(S4)}$$

The effective Hamiltonian of the *PTX* system can be defined as: $H'_{\text{eff}} = i\mathcal{L}'$, which has a non-Hermitian form $H'^{\dagger}_{\text{eff}} \neq H'_{\text{eff}}$. The effective Hamiltonian is symmetric with respect to the combined $\mathcal{PTX}$ transformation, i.e., $[\mathcal{PTX}, H'_{\text{eff}}] = 0$, with $\mathcal{P}$ and $\mathcal{T}$ transformations are consistent with those of *PT*-symmetric systems, while

$$\mathcal{X} = \mathbf{I} \otimes x_0 \text{ and } x_0 = \begin{pmatrix} x^{-1/2} & 0 & 0 \\ 0 & x^{-1/2} & 0 \\ 0 & 0 & x^{1/2} \end{pmatrix}. \quad \text{(S5)}$$

The effective Hamiltonian and eigenmodes of the *PTX* system are related to those of *PT* system through the similarity transformation $H'_{\text{eff}} = S^{-1} H_{\text{eff}} S$ and $\mathbf{\Psi}' = S^{-1}\mathbf{\Psi}$, where S is an invertible 6×6 matrix,

$$S = \mathbf{I} \otimes \zeta \text{ and } \zeta = \begin{pmatrix} x^{-1/2} & 0 & 0 \\ 0 & x^{-1/2} & 0 \\ 0 & 0 & 1 \end{pmatrix}. \quad \text{(S6)}$$

We note that in either case, the eigenfrequencies of the system can be found by solving Eq. (S1) after the substitution of $q_n = A_n e^{i\omega\tau}$, or the direct diagonalization of the matrix $\mathcal{L}$ or $\mathcal{L}'$, i.e., $\left|H_{\text{eff}} - \omega\mathbf{I}\right| = 0$ or $\left|H_{\text{eff}}{}' - \omega\mathbf{I}\right| = 0$, given by

$$\omega_1 = \omega_0 \sqrt{\frac{2\gamma^2 - 1 - \sqrt{1 - 4\gamma^2 + 8\gamma^4\kappa^2}}{2\gamma^2\left(1 - 2\kappa^2\right)}}, \quad \text{(S7A)}$$

$$\omega_2 = \omega_0, \quad \text{(S7B)}$$

$$\omega_3 = \omega_0 \sqrt{\frac{2\gamma^2 - 1 + \sqrt{1 - 4\gamma^2 + 8\gamma^4\kappa^2}}{2\gamma^2\left(1 - 2\kappa^2\right)}}. \tag{S7C}$$

To avoid redundancy, our analysis focuses mainly on positive eigenfrequencies, and the negative counterparts which lack significant implementations in real physical contexts are excluded. Notably, the three eigenfrequencies the system is independent of the scaling factor *x*, indicating that *PTX* and *PT* systems share the same eigenfrequencies. This allows utilizing a single reader to retrieve information from multiple sensing resonators, while offering flexibility in sensor design. Moreover, there exists an exceptional point (EP), where the eigenfrequencies bifurcate, given by

$$\gamma_{\mathrm{EP}} = \frac{\sqrt{1 + \sqrt{1 - 2\kappa^2}}}{2\kappa}. \tag{S8}$$

The EP divides the system into the broken *PT*-symmetric phase ($\gamma < \gamma_{\mathrm{EP}}$) and the exact *PT*-symmetric phase ($\gamma > \gamma_{\mathrm{EP}}$). The three eigenfrequencies, as seen in Fig. 2A and fig. S5, are purely real in the exact *PT*-symmetric phase ($\gamma > \gamma_{\mathrm{EP}}$), appearing as the resonant frequencies or minima in the reflection spectra.

Upon simultaneous resistive and capacitive perturbations, the generalized *PT*-symmetric conditions may be slightly broken. Thus, we consider a small unbalanced resistive perturbation at the sensor, i.e., $xR_3 = (1+\tau)\,|-R\,|$ and $\tau \ll 1$, the eigenfrequencies of the third-order system can be approximately expressed as:

$$\omega_1' \approx \omega_0 \sqrt{\frac{2\gamma^2 - 1 - \tau - \sqrt{(1+\tau)^2 - 4\gamma^2(1+\tau) + 8\gamma^4\kappa^2}}{2\gamma^2\left(1 - 2\kappa^2\right)}}, \tag{S9A}$$

$$\omega_2' \approx \omega_0 (= 1/\sqrt{LC}), \tag{S9B}$$

$$\omega_3' \approx \omega_0 \sqrt{\frac{2\gamma^2 - 1 - \tau + \sqrt{(1+\tau)^2 - 4\gamma^2(1+\tau) + 8\gamma^4\kappa^2}}{2\gamma^2\left(1 - 2\kappa^2\right)}}. \tag{S9C}$$

Notably, when $\tau = 0$, Eq. (S9) is equivalent to Eq. (S7), indicating that the system satisfies the generalized *PT*-symmetry. Equation (S9) states that with slightly unbalanced resistance at the sensor, ω_1 and ω_3 are influenced by the resistive perturbation while ω_2 remains unchanged. As a consequence, no matter that the system is symmetric or slightly asymmetric in resistance, ω_2 is only related to inductance and capacitance values, and is independent of γ and κ. The capacitive perturbation can be detected by tracking ω_2, even when a slight resistive perturbation is unknown and unbalanced.

Note S2. Theoretical calculation of the reflection spectrum of the generalized high-order EP-based monitoring system

For both *PT*- and *PTX*-symmetric conditions, the sensing information is encoded in the reflection coefficient (S_{11}) measured at the reader terminal, which can be written by

$$S_{11} = \frac{Z_{\mathrm{in}} - Z_0}{Z_{\mathrm{in}} - Z_0}, \tag{S10}$$

where Z_{in} is the input impedance looking into the "–RLC" tank from the terminal and Z_0 is the characteristic impedance of the terminal ($Z_0 = 50\ \Omega$). The input impedance of the EP monitoring system can be derived as

$$Z_{\mathrm{in}} = Z_0 \frac{-i(\eta-1)\left(\omega^2-1\right)\omega^2 + \gamma\omega\left[\left(\kappa^2-1\right)\omega^4 + 2\omega^2 - 1\right] + i\gamma^2\eta\left(\omega^2-1\right)\left[\left(2\kappa^2-1\right)\omega^4 + 2\omega^2 - 1\right]}{i\omega^2\left(\omega^2-1\right) + \gamma\omega\left[\left(\kappa^2-1\right)\omega^4 + 2\omega^2 - 1\right]}, \tag{S11}$$

where $\eta\ (= R/Z_0)$ is the impedance normalization factor. By casting Eq. (S11) into Eq. (S10), we obtain

$$S_{11} = \frac{\eta\left(\omega^2-1\right)\left[-\omega^2 + \gamma^2\left(\left(2\kappa^2-1\right)\omega^4 + 2\omega^2 - 1\right)\right]}{-\left[(\eta-2)\omega^2\left(\omega^2-1\right)\right] - 2i\gamma\omega\left[\left(\kappa^2-1\right)\omega^4 + 2\omega^2 - 1\right] + \gamma^2\eta\left(\omega^2-1\right)\left[\left(2\kappa^2-1\right)\omega^4 + 2\omega^2 - 1\right]}. \tag{S12}$$

From Eqs. (S11) and (S12), it is evident that the *PT*- and *PTX*-symmetric systems share the same input impedance and reflection coefficient, independent of x. Upon the system operates at its resonant frequency, a perfect impedance matching is obtained, i.e., $Z_{\mathrm{in}} = Z_0$, leading to a zero for S_{11} and a sharp resonance dip in the reflection spectrum.

Note S3. Theoretical calculation of conventional "LC" resonator-based sensors and standard EP monitoring systems

Conventional "LC" resonator-based sensors. The eigenfrequency of the conventional "LC" resonator-based sensing system is given by

$$\omega^{(\mathrm{LC})} = \frac{i\left(1+\sqrt{1+4\gamma^2\left(\kappa^2-1\right)}\right)}{2\gamma\left(\kappa^2-1\right)}. \tag{S13}$$

The eigenfrequency of the "LC" sensing system is complex, with its real part equivalent to the resonance frequency. The resonant frequency of the "LC" sensing system can be approximated as

$$\omega^{(\mathrm{LC})} \approx \frac{1}{\sqrt{LC}}. \tag{S14}$$

It is noted from Eq. (S14) that the "LC" sensing system exhibit single resonant frequency which is related to the inducatance and capacitance values of the system. Given that the inductors are generally kept unchanged for resonator-based sensors, the "LC" sensing system can only monitor a singluar capacitive variation, uncapable of resistive or multi-parameter monitoring. The input impedance and reflection coefficient of the "LC" sensing system is given by

$$Z_{\mathrm{in}}^{(\mathrm{LC})} = R_L + Z_0\frac{\gamma\eta\omega^2 - i\gamma^2\eta\omega\left[\left(\kappa^2-1\right)\omega^2+1\right]}{-i\omega+\gamma\left(\omega^2-1\right)}, \tag{S15}$$

$$S_{11}^{(\mathrm{LC})} = \frac{R_L\left[\gamma\left(\omega^2-1\right)-i\omega\right]+Z_0\left[i\omega+\gamma+\gamma\left(\eta-1\right)\omega^2-i\gamma^2\eta\omega\left(\left(\kappa^2-1\right)\omega^2+1\right)\right]}{R_L\left[\gamma\left(\omega^2-1\right)-i\omega\right]-Z_0\left[i\omega+\gamma-\gamma\left(\eta+1\right)\omega^2+i\gamma^2\eta\omega\left(\left(\kappa^2-1\right)\omega^2+1\right)\right]}. \tag{S16}$$

Unlike EP systems where resonant frequencies indicate zeros in the reflection spectrum, the "LC" system's resonant frequency appears as the minima but not zero of the reflection coefficient, corresponding to relatively low quality factor and sensitivity (Fig. 3D).

Standard EP sensing systems. The standard (second-order) EP system comprises a pair of inductively coupled "–RLC" and "RLC" resonators, which serve as the reader and sensor in a sensing system, respectively. The eigenfrequencies of the standard EP system can similarly be derived from the eigenvalue equation $\left|H_{\mathrm{eff}}-\omega\mathbf{I}\right|=0,$ written as

$$\omega_1^{(\mathrm{standard})} = \omega_0\sqrt{\frac{2\gamma^2-1-\sqrt{1-4\gamma^2+4\gamma^4\kappa^2}}{2\gamma^2\left(1-\kappa^2\right)}}, \tag{S17A}$$

$$\omega_2^{(\mathrm{standard})} = \omega_0\sqrt{\frac{2\gamma^2-1+\sqrt{1-4\gamma^2+4\gamma^4\kappa^2}}{2\gamma^2\left(1-\kappa^2\right)}}. \tag{S17B}$$

Both eigenfrequencies are related to γ and κ values, bifurcating at the EP, given by

$$\gamma_{\mathrm{EP}}^{(standard)} = \frac{1}{\kappa}\sqrt{\frac{1+\sqrt{1-\kappa^2}}{2}}. \tag{S18}$$

As a result, the standard EP system is unable to distinguish resistive and capacitive perturbations. The input impedance and reflection coefficient of the standard *PT* system can be written as

$$Z_{\text{in}}^{(\text{standard})} = Z_0 \frac{i(\eta-1)\omega^2 + \gamma\omega(\omega^2-1) - i\gamma^2\eta\left[(\kappa^2-1)\omega^4 + 2\omega^2 - 1\right]}{-i\omega^2 + \gamma\omega(\omega^2-1)}, \tag{S19}$$

$$S_{11}^{(\text{standard})} = \frac{-\eta\omega^2 + \gamma^2\eta\left[(\kappa^2-1)\omega^4 + 2\omega^2 - 1\right]}{-(\eta-2)\omega^2 + 2i\gamma\omega(\omega^2-1) + \gamma^2\eta\left[(\kappa^2-1)\omega^4 + 2\omega^2 - 1\right]}. \tag{S20}$$

Note S4. Noise analysis of the third-order EP-based monitoring system

Noise in electromagnetic systems can arise from shot noise, flicker noise, thermal noise, and quantum noise. Quantum noise, originating from the quantization of charged carriers and photons, is relevant in optical and photonic systems but negligible in our radio-frequency platform. Shot noise and flicker noise occur in solid-state and vacuum devices and dominate only at low frequencies (1 Hz–1 MHz). The dominant noise source in our work is thermal noise, which originates from the thermal agitation of bounded charges in resistors and directly contributes to resonance frequency fluctuations.

According to Planck's black body radiation law, electrons in a real resistor are in random motion, producing small random voltage fluctuations with zero mean but finite root mean square (RMS) value:

$$\overline{V}_{\text{noise}} = \sqrt{\frac{4hfBR}{e^{hf/kT}-1}}, \tag{S21}$$

where h represents Planck's constant, f and B are the center frequency and bandwidth, k is the Boltzmann's constant, R is the resistance value, and T denotes the temperature. In the radio-frequency regime, we can take the approximation $hf \ll kT$ and the above equation can therefore be simplified to $\overline{V}_{\text{noise}} = \sqrt{4kTBR}$. This indicates that the maximum voltage fluctuation due to thermal noise is $\pm\sqrt{8kTBR}$. Thus, a non-ideal noisy resistor can modeled as an ideal resistor R in series with a noisy resistor R' $(\propto \sqrt{8kTBR})$. Defining a fluctuation parameter $\Delta = R'/R$, the resonance frequency under maximum noise can be written as

$$\omega'_{1,3} = \omega_0 \sqrt{\frac{2\gamma^2(1+\Delta)^2 - 1 \mp \sqrt{1-4\gamma^2(1+\Delta)^2 + 8\gamma^4(1+\Delta)^4\kappa^2}}{2\gamma^2(1+\Delta)^2\left(1-2\kappa^2\right)}}. \tag{S22}$$

The second resonant frequency can be derived in a similar manner, accounting for thermal noise from non-ideal conductors on the circuit board. Here, our analysis focused on the third resonance frequency, as it is most susceptible to noise. Considering the system operating close to the EP ($\Delta\gamma = 0.015$, $\kappa = 0.5$, $f_0 = \omega_0 / 2\pi = 45.34$ MHz), the maximum frequency fluctuation is predicted as $\Delta f_3 = (\omega'_3 - \omega_3)/2\pi = 24.19$ kHz with a bandwidth of 60 MHz.

Experimentally, we measured frequency drifts under the these condition. As shown in fig. S9A, the observed maximum fluctuations were ±46.20 kHz and ±69.55 kHz for the second and third resonant frequencies, respectively. These results are in good agreement with theoretical predictions, with small additional contributions from cables, adaptors, and equipment. To further quantify performance, we evaluate stability and resolution using Allan deviation, defined as

$$\sigma(\tau) = \frac{1}{\overline{f}} \sqrt{\frac{1}{2(M-1)} \sum_{n=1}^{M-1} (\overline{\Delta f_{n+1}} - \overline{\Delta f_n})^2}, \tag{S23}$$

where τ is the sampling time, $\overline{f}$ is the average resonance frequency, M is the number of samples, and $\overline{\Delta f}$ is the average instantaneous frequency deviation in interval [$n\tau$, $(n+1)\tau$]. As shown in fig. S9B, the second and third resonant frequencies exhibit Allan deviations of 3.95×10^{-4} and 4.48×10^{-4}, respectively, with a sampling time of 0.5 s. These small deviation

values confirm that our system maintains high stability, even in close proximity to the EP. At larger detuning from the EP or with longer sampling time, the Allan deviation decreases further, demonstrating even greater stability. This excellent stability arises primarily from the use of fully passive components (resistors, inductors, and capacitors) and the complete elimination of active components, which renders the system unconditionally stable even in noisy environments.

Further, we quantify the resolution of our system, expressed as

$$\text{Resolutoin} = \frac{\sigma(\tau)\overline{f}}{s}, \tag{S24}$$

where $\sigma(\tau)$ is the Allan deviation, $\overline{f}$ is the average resonance frequency, and s is the sensitivity defined in Fig. 3C. The absolute Allan deviation $\sigma(\tau)\overline{f}$ are measured as 17.41 kHz and 24.32 kHz for the second and third resonance frequencies, respectively, with a sampling time of 0.5 s. The corresponding resolutions for capacitive and resistive perturbations, derived from shifts in f_2 and f_3, are 0.0005 and 0.0002, respectively. The smaller resolution for the third resonance arises from frequency splitting at the EP. These resolution values are sufficient to detect variations in health-related biomarkers, as validated in our sweat monitoring experiments.

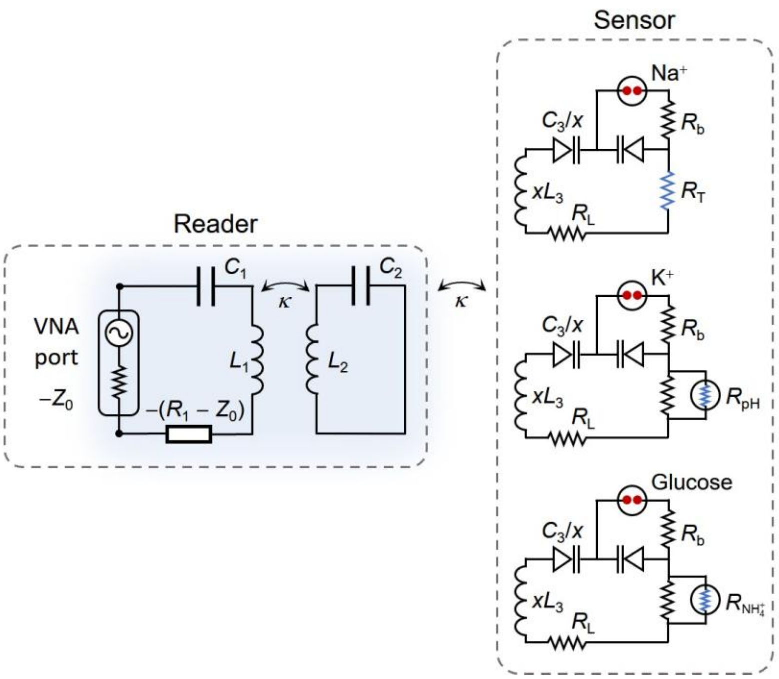

Fig. S1. Circuit schematic of the high-order EP-based system for perspiration monitoring. Here, R_L is the resistance of the coil and conductive traces, R_b = 100 Ω is the resistance of current-limiting resistors employed to protect the varactor diodes, R_T, R_{pH}, and $R_{NH_4^+}$ are the resistance of the thermistor, pH-sensitive resistor, NH_4^+-sensitive resistor, respectively. A 121-Ω resistor and a 330-Ω resistor are in parallel with the pH- and NH_4^+-sensitive resistor, respectively, to make the system satisfy the generalized *PT*-symmetric conditions. For T-Na^+ resonator, the scaling factor x = 1, the equivalent resistance $xR_3 = R_T + R_L$. For the pH-K^+ resonator, the scaling factor x = 3.5, the equivalent resistance $xR_3 = R_{pH} \parallel 121\ \Omega + R_L$. For NH_4^+-Glucose resonator, the scaling factor x = 3.5, the equivalent resistance $xR_3 = R_{NH_4^+} \parallel 330\ \Omega + R_L$.

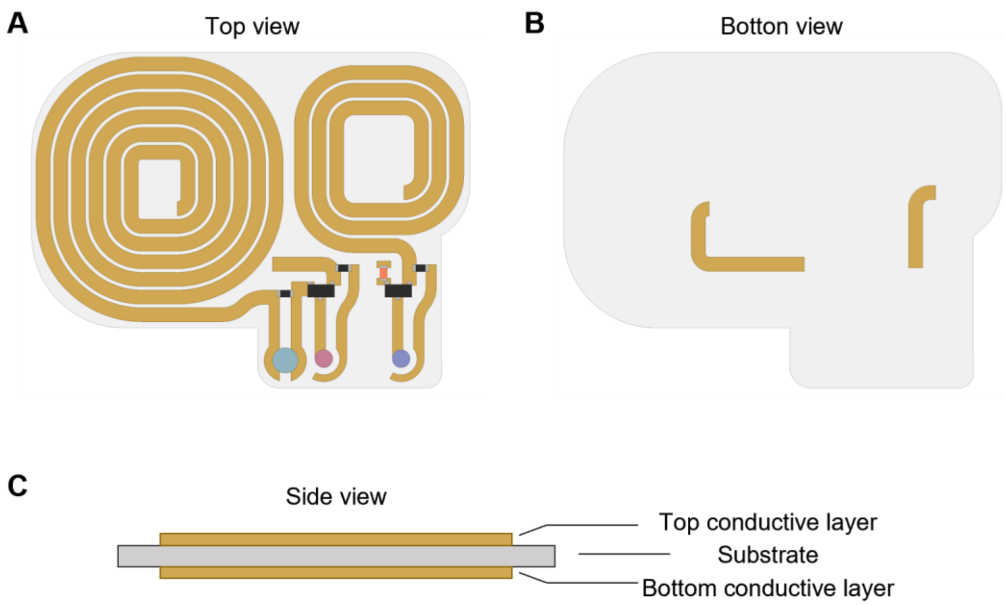

Fig. S2. Top (A), bottom (B), and side views (C) of the wearable sensor. The sensor consists of a substrate layer sandwiched by top and bottom conductive layers.

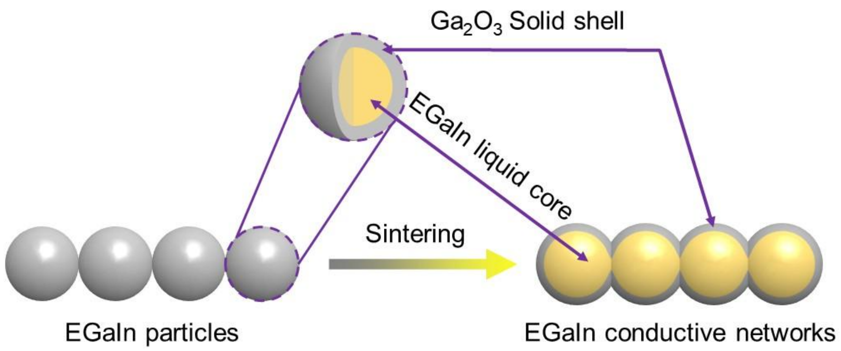

Fig. S3. Schematic illustration of the sintering progress of the EGaIn composites. Before sintering, EGaIn particles are encapsulated by Ga_2O_3 (gallium oxide) oxidation layer. During the sintering process, the mechanical sintering disrupts the Ga_2O_3 oxidation layer, allowing the EGaIn particles to connect and form a conductive network.

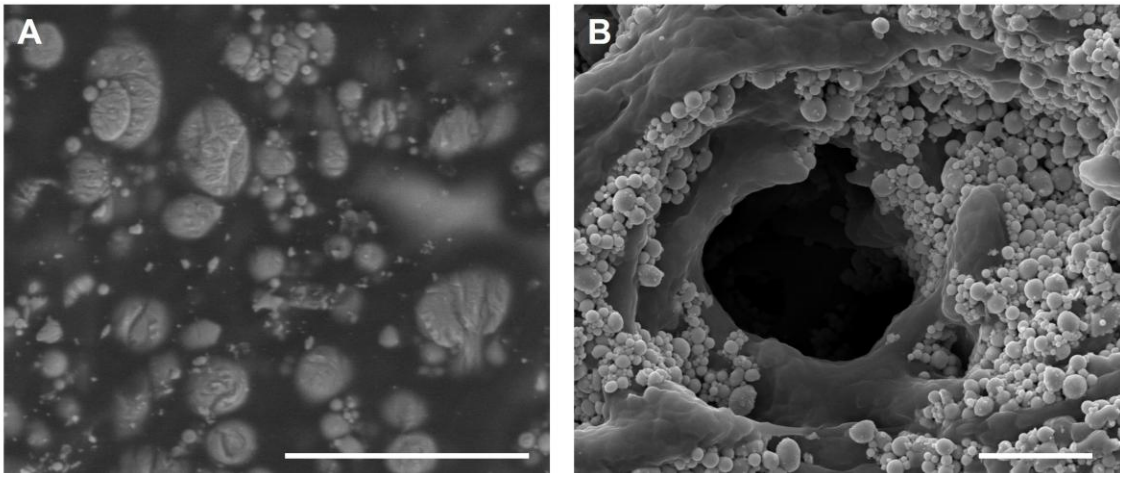

Fig. S4. SEM images of nonporous (A) and porous (B) EGaIn composites before sintering. (A) shows EGaIn particles distributed within the polyurethane (PU) elastomer matrix. A higher filling ratio is necessary for these particles to form percolation networks. (B) clearly reveals the porous structure where EGaIn particles self-organize on the surfaces of the pores. Scale bar, 5 µm.

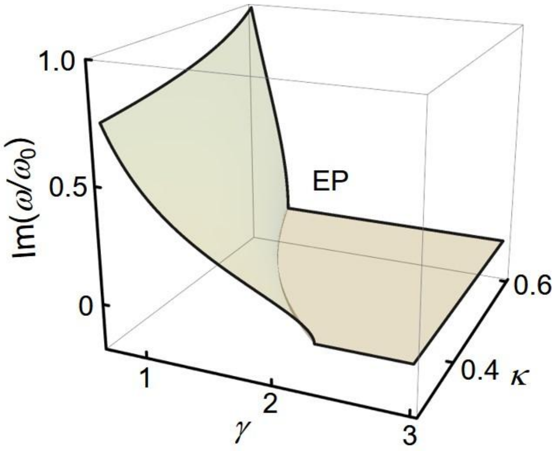

Fig. S5. Imaginary part of eigenfrequencies as a function of effective *Q*-factor γ and coupling strength κ. The imaginary part becomes zero when $\gamma > \gamma_{\mathrm{EP}}$, indicating a purely real eigenfrequency.

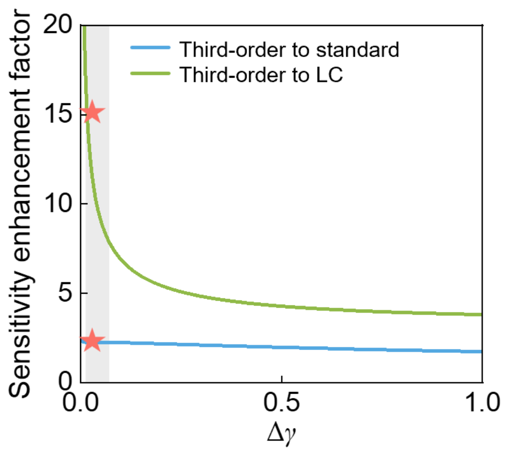

Fig. S6. Sensitivity improvement of the third-order EP system compared with the standard EP and "LC" systems. The sensitivity improvement factor is defined as the ratio of the sensitivity of the third-order EP system to that of standard EP or "LC" system. The grey-shaded region ($0.015 < \Delta\gamma < 0.05$) represents operation near the EP, where the averaged sensitivity enhancement factors are 2.26 and 11.17 relative to the standard EP and "LC" systems, respectively. The red stars mark maximum experimentally achieved enhancement, corresponding to factors of 2.27 and 15.35.

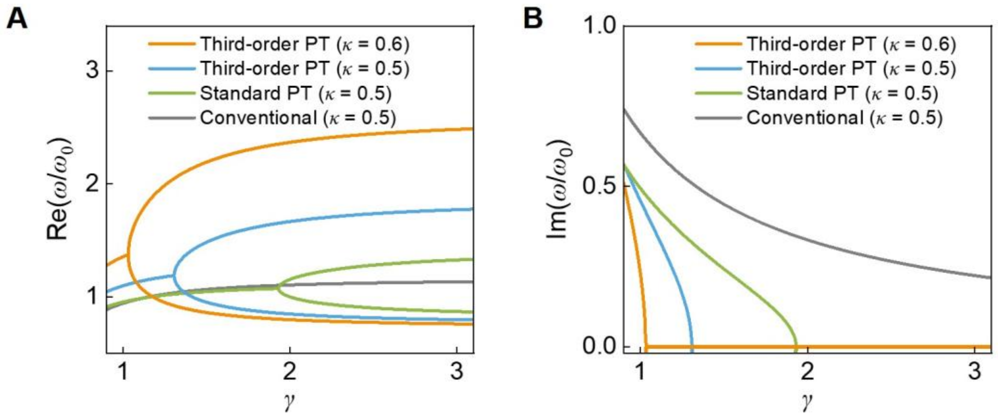

Fig. S7. Real (A) and imaginary (B) parts of eigenfrequencies for the third-order EP, standard EP, and conventional "LC" sensing systems. The third-order EP system demonstrates much improved sensitivity, i.e., the slope of Re (ω/ω_0) versus γ, which can be further enhanced by employing high coupling strength.

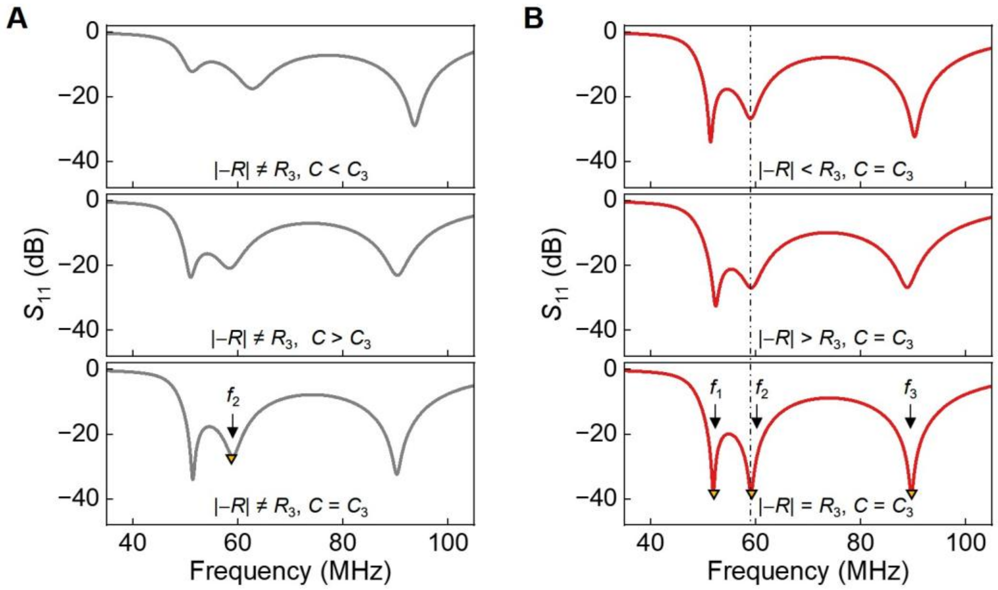

Fig. S8. Measurement of equivalent resistance *R_3* and capacitance *C_3* in the sensing resonator. (A) Measured reflection spectra when both R_3 and C_3 are unknown, and one first tries to determine C_3, and (B) after C_3 is known, one tries to find the exact value of R_3. Here, we consider $x = 1$ for simplicity. During the measurement, a reflection minimum was first found at f_2 by tuning the capacitance of the reader, the resonance frequency f_2 is used as a sign of balance in capacitance (i.e., $C = C_3$). Subsequently, three reflection dips were acquired by adjusting the resistance of the reader until the generalized *PT*-symmetric condition is achieved (i.e., $|-R| = R_3$, $C = C_3$). By this simple, two-step tracking of resonance frequencies of the third-order EP system, R_3 and C_3 values can be obtained by a single resonator with high accuracy.

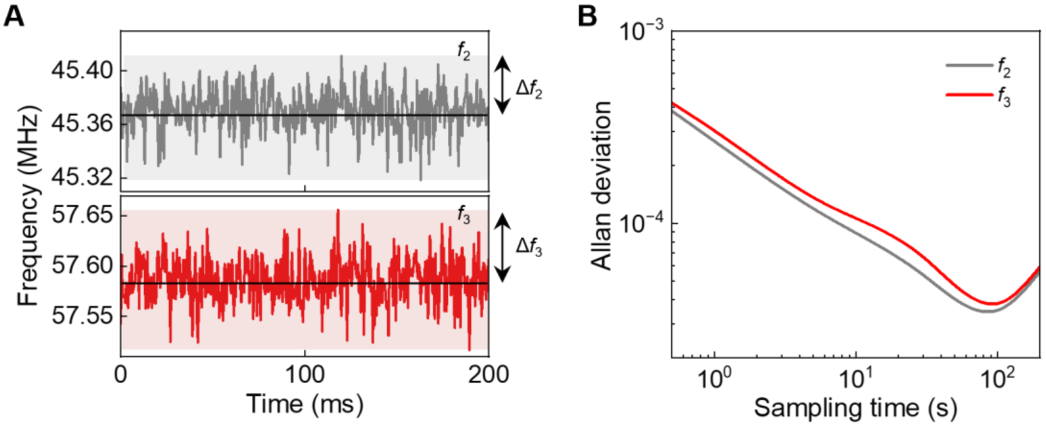

Fig. S9. Noise analysis of the third-order EP sensing system. (A) presents the continuously recorded second and third resonance frequencies. (B) shows the Allan deviation of the second and third resonance frequencies with sampling time.

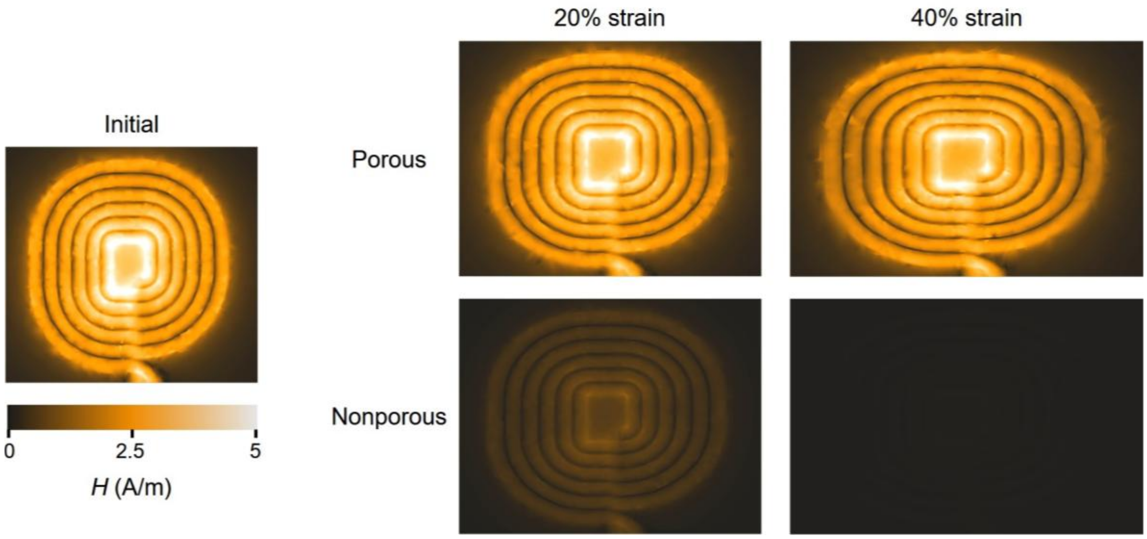

Fig. S10. Magnetic field distributions of the 770 nH coil inductors made of nonporous and porous LM composites under strain. The magnetic field distributions were attainted via Ansys Maxwell, a comprehensive electromagnetic field simulation software.

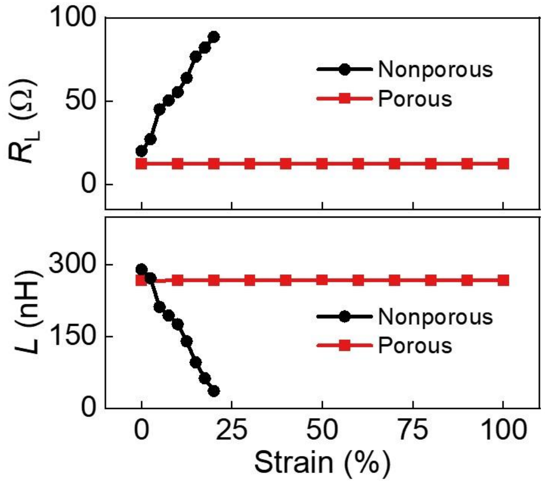

Fig. S11. Resistance and inductance of the 220 nH coil inductor under uniaxial strain. Here, we compare the coils made of nonporous and porous LM composites, demonstrating the superior reliability of the PLMC upon stretching.

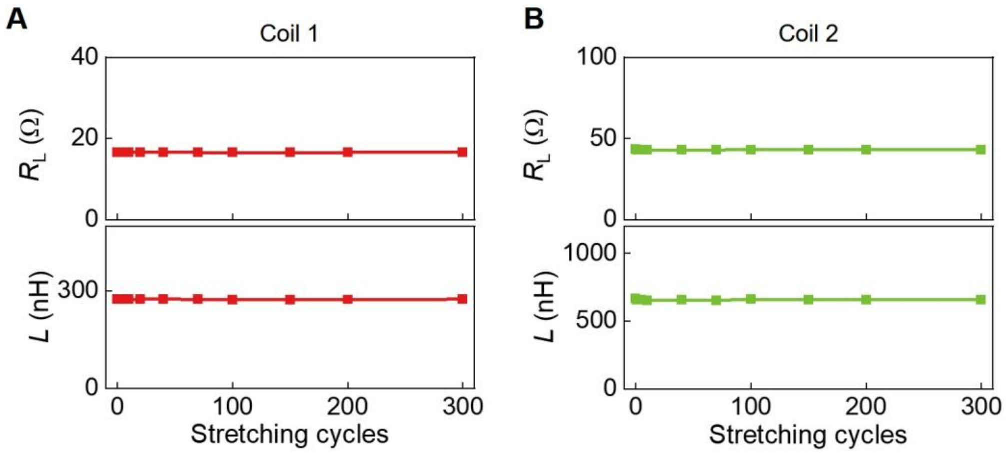

Fig. S12. Resistance and inductance of the 220 nH (coil 1) and 770 nH (coil 2) inductors under repeated stretching (100% maximum strain).

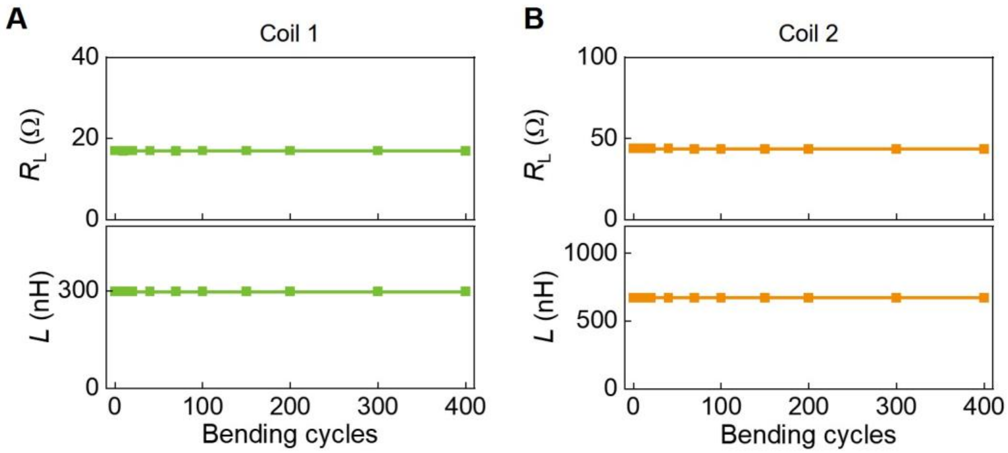

Fig. S13. Resistance and inductance of the 220 nH (coil 1) and 770 nH (coil 2) inductors under repeated bending (1 mm bending radius).

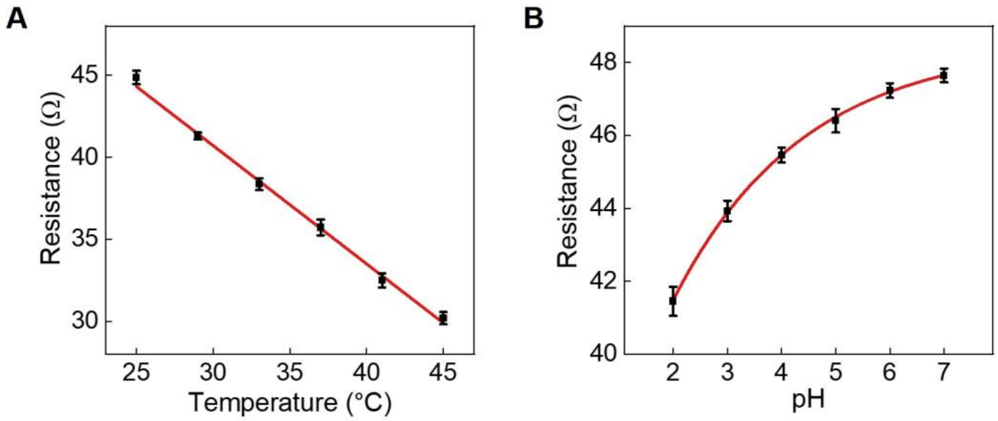

Fig. S14. Equivalent resistance R_3 of the sensing resonator when a thermistor and a pH-sensitive resistor are respectively employed for temperature (A) and pH (B) monitoring. Here, the resistance value considers the conductive traces, with details presented in fig. S1.

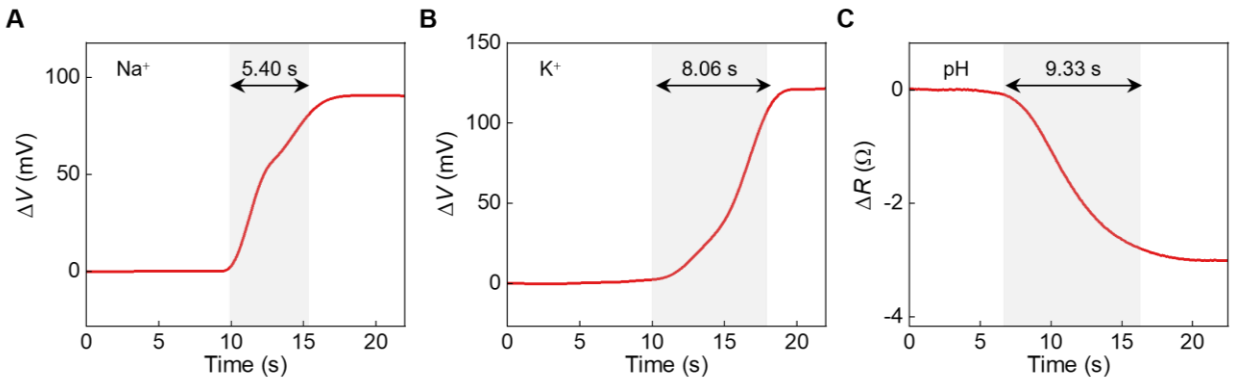

Fig. S15. Response of ion sensors. A two-order-of-magnitude change in Na^+, K^+ concentration and pH results in response times of and 5.40 s, 8.06 s, and 9.33 s, respectively, to reach 90% of the stable voltage.

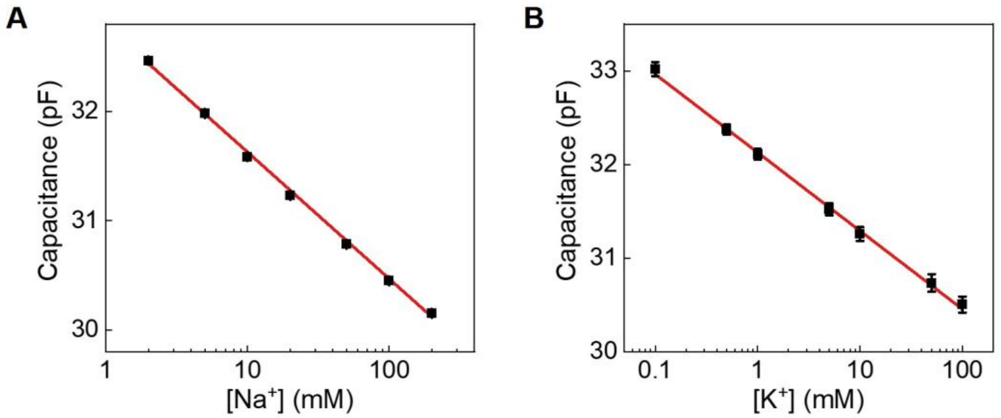

Fig. S16. Equivalent capacitance C_3 of the sensing resonator when custom-made electrodes are employed for [Na^+] (A) and [K^+] (B) monitoring. The electrodes are connected to varactors for translating the electrical potential between electrodes to capacitance. Details are provided in fig. S1 and Materials and Methods in the main text.

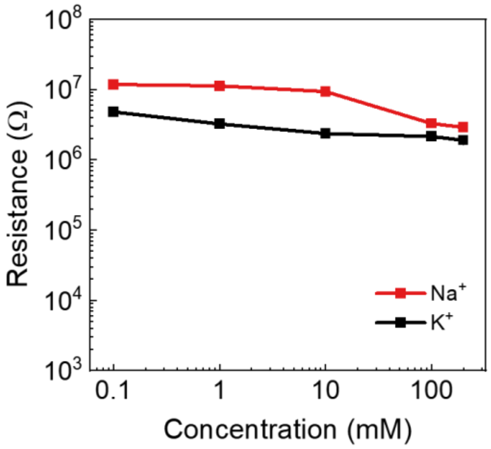

Fig. S17. Relationship between resistance and ion concentration. The result indicates that when the concentration increases from 0.1 mM to 200 mM, the resistance of the Na^+ sensor decreases from 11.8 MΩ to 2.9 MΩ, while the resistance of the K^+ sensor decreases from 4.8 MΩ to 1.9 MΩ.

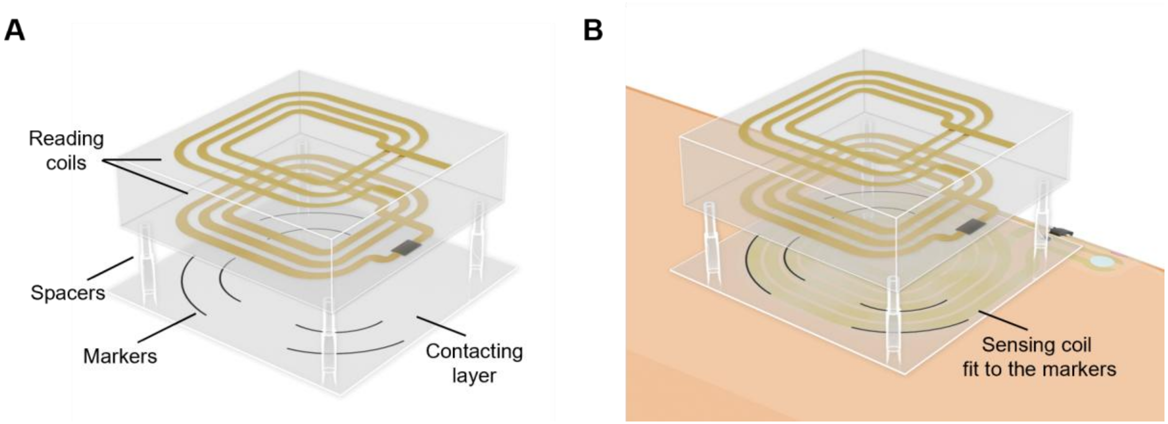

Fig. S18. Illustrations of the reader for precise coil alignment. Spacers and markers are employed in the reader to fix the relative reader-sensor position without sacrificing user convenience and wearability.

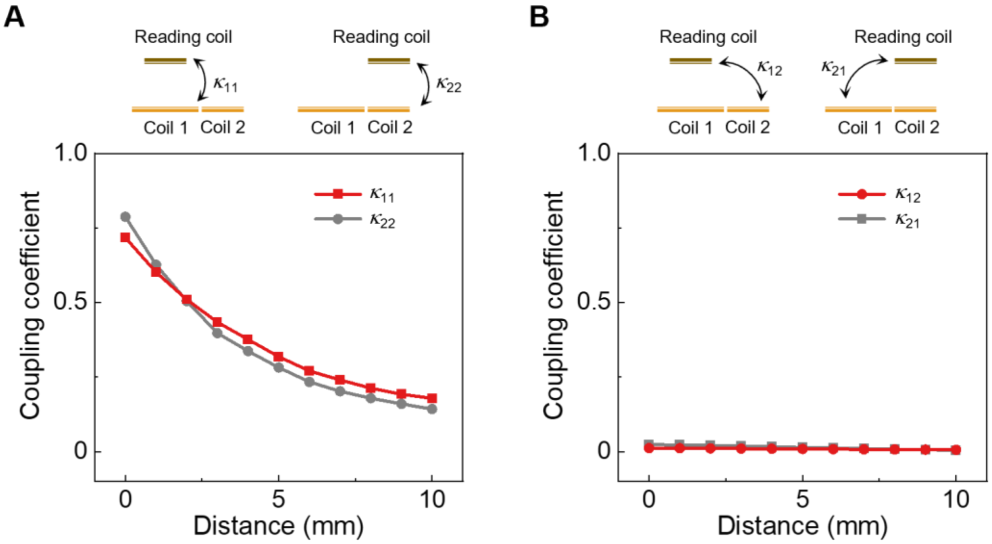

Fig. S19. Coupling coefficients between the reader coil and two sensor coils (A) and between the reader coil and non-targeted sensor coil (B). When coil distance increases from 0 to 10 mm, the coupling coefficient between the reader and the monitored sensor varies from ~0.75 to ~0.15, while those between the reader and the non-targeted sensor consistently remains below 0.02.

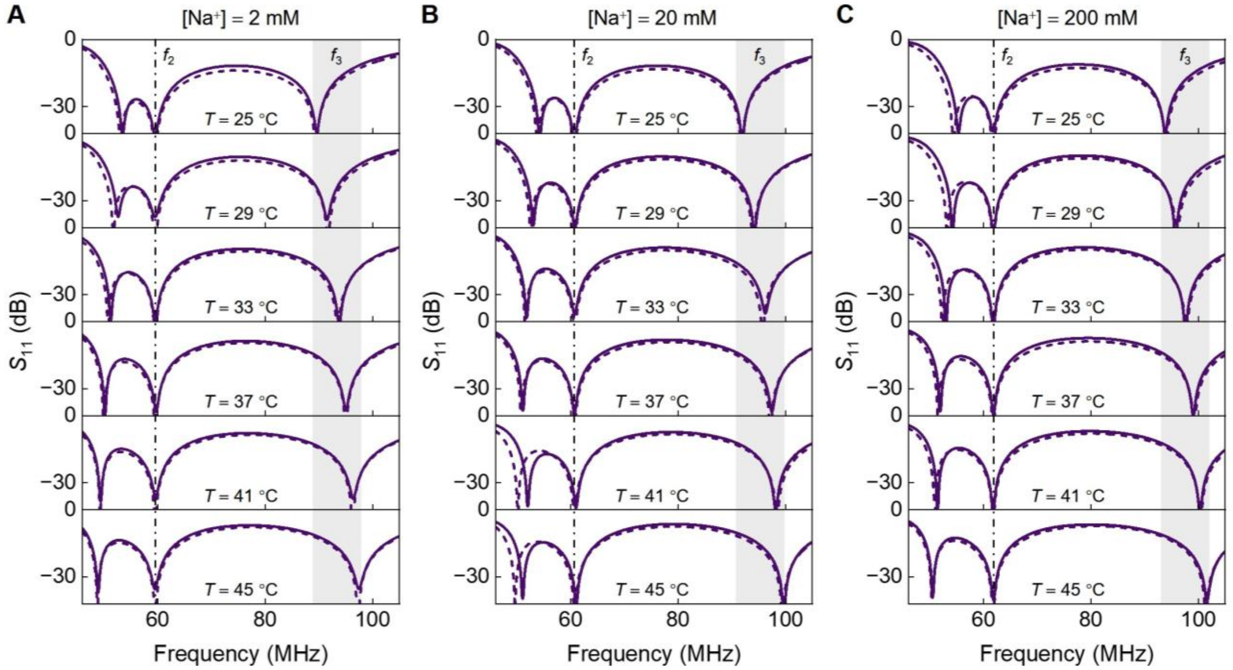

Fig. S20. Measured reflection spectra of the sweat monitoring system with various temperature conditions. The third resonant frequency can notably shift with temperature variations, indicating high sensitivity.

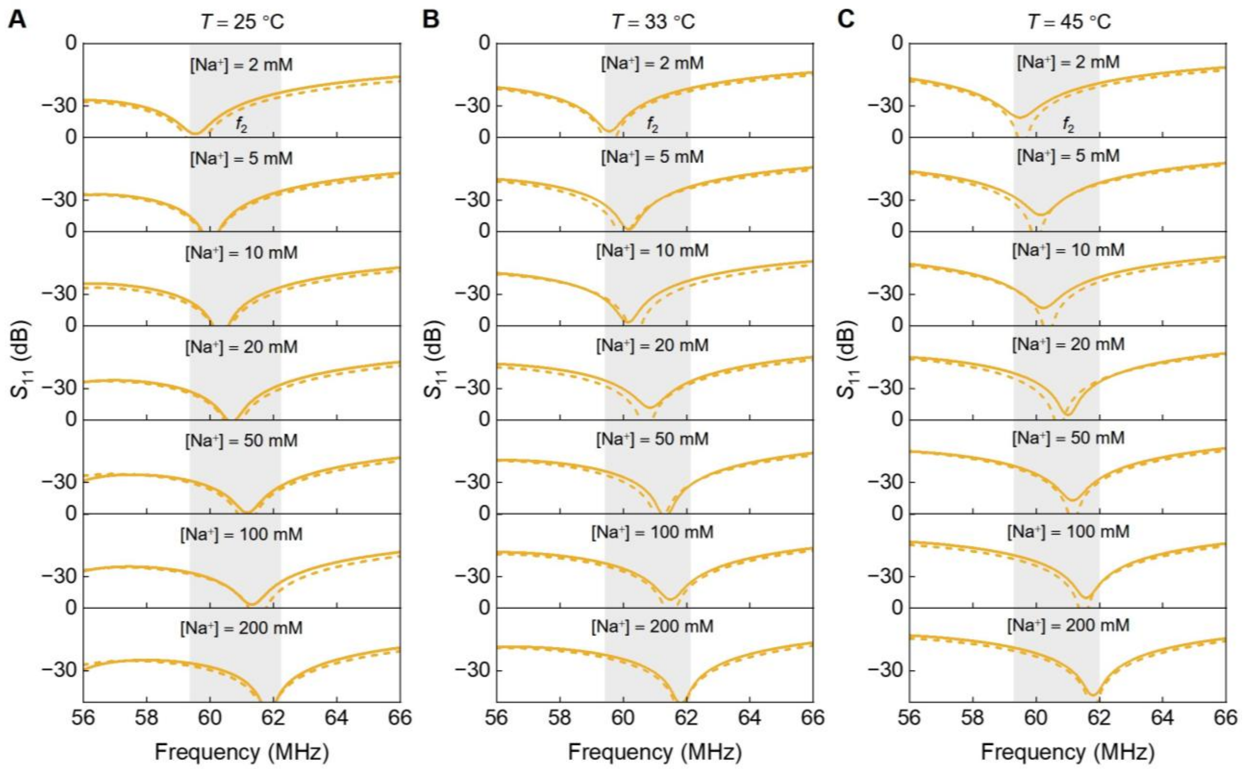

Fig. S21. Measured reflection spectra of the sweat monitoring system with various Na^+ concentrations. The second resonant frequency is responsive to Na^+ concentrations, regardless of temperature conditions.

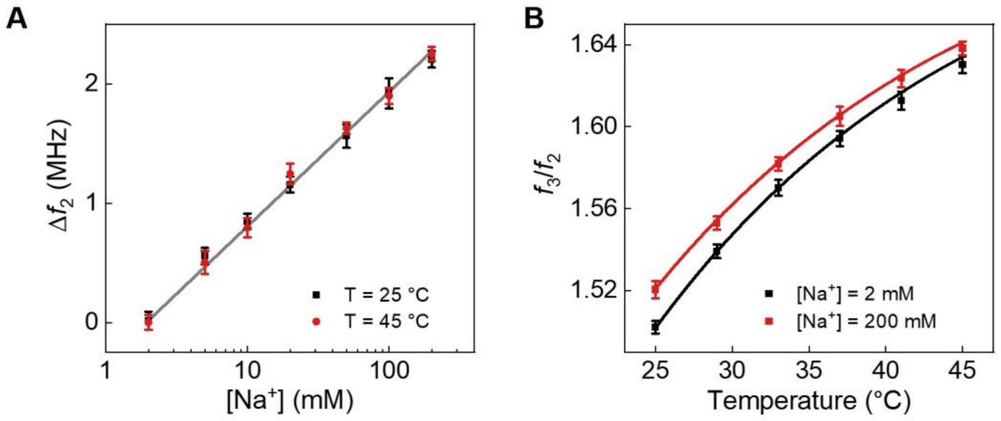

Fig. S22. Monitoring of temperature and Na^+ concentration. (A) The shift in the second resonant frequency f_2 with respect to Na^+ concentration, independent of temperature conditions. (B) The frequency ratio f_3/f_2 as a function of temperature, demonstrating high sensitivity.

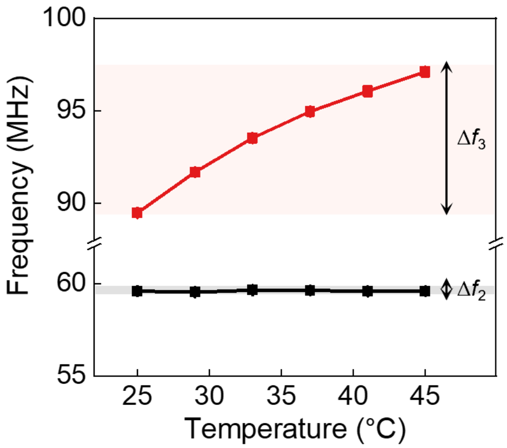

Fig. S23. Shifts of the second and third resonance frequencies with increasing temperature. The third resonance frequency shifts 7.62 MHz while the second resonance frequency fluctuates only 90.02 kHz (~1.2% compared to changes in the third-resonance frequency).

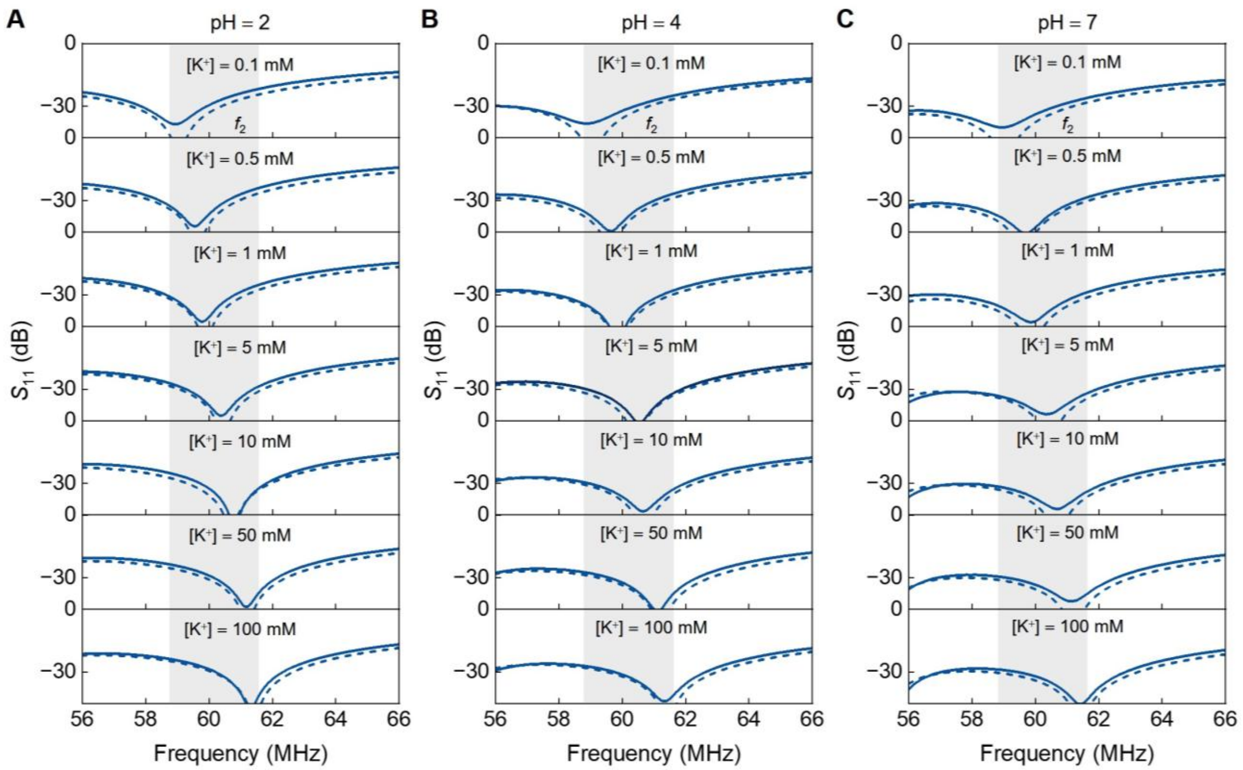

Fig. S24. Measured reflection spectra of the sweat monitoring system with various K^+ concentrations. The second resonant frequency is responsive to K^+ concentrations, independent of pH level.

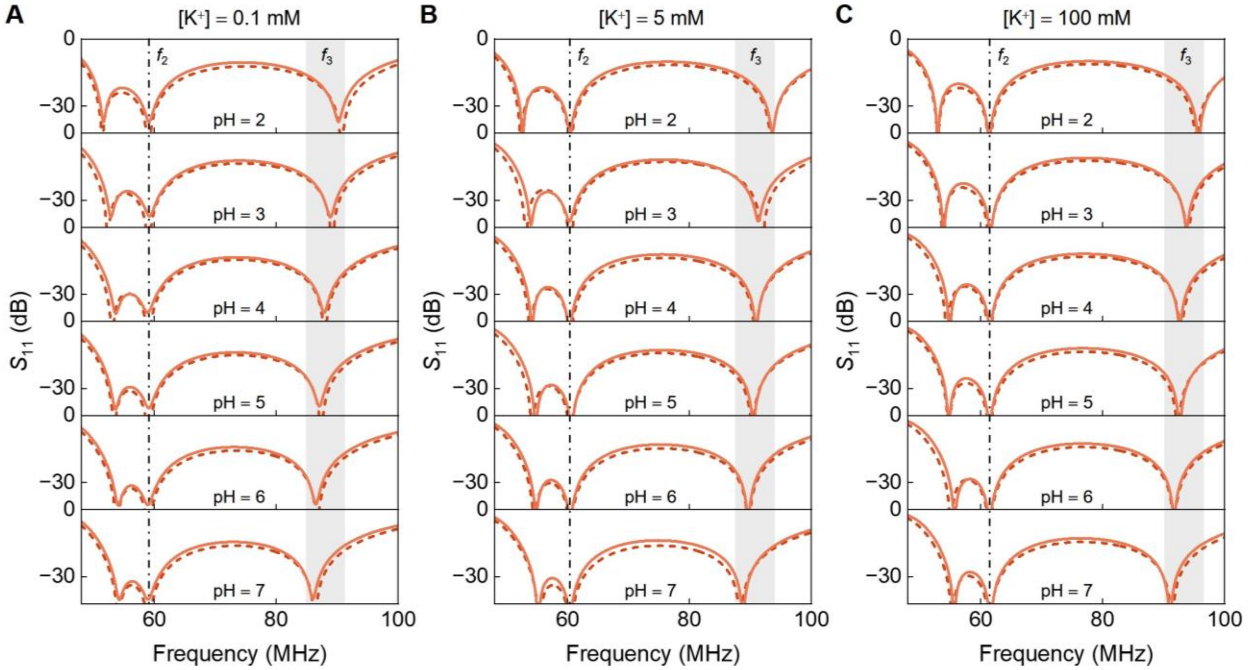

Fig. S25. Measured reflection spectra of the sweat monitoring system with various pH. The third resonant frequency can notably shift with pH, while the second resonant frequency is unchanged.

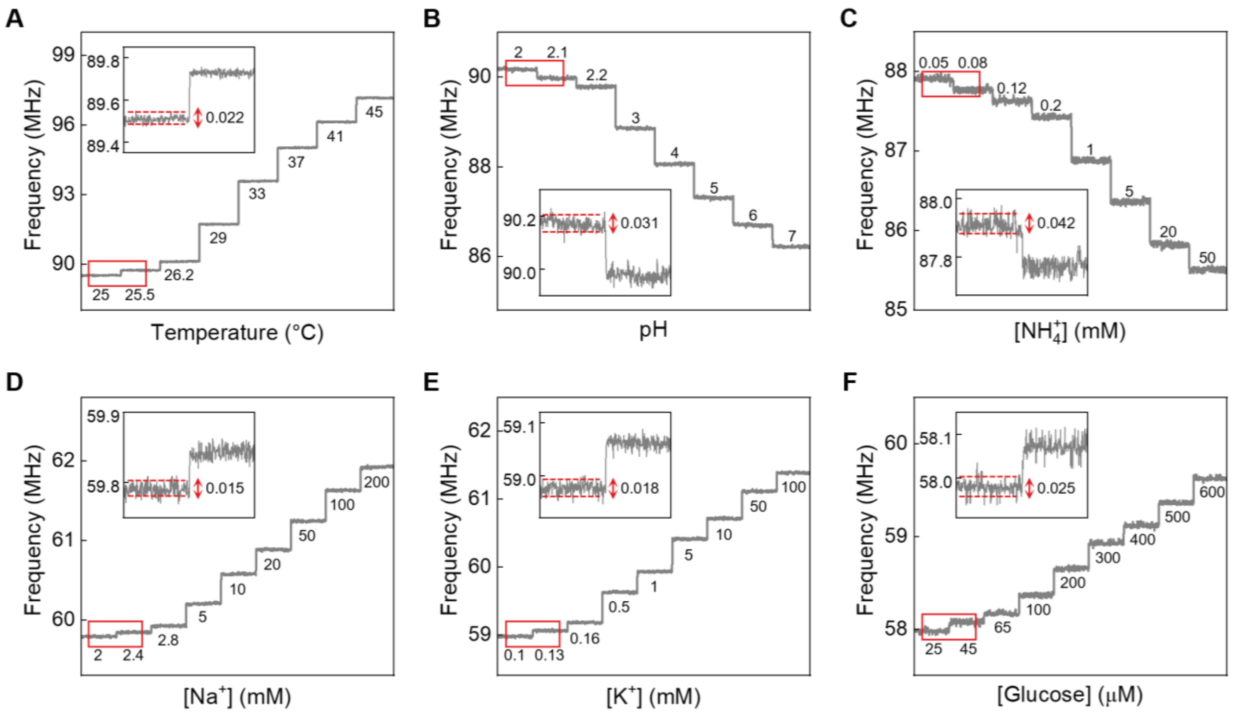

G

Biomarker	Sensitivity	Absolute Allan deviation	Resolution	Q-factor	SNR
T	0.39 MHz/℃ at [Na⁺] = 2 mM	11.16 kHz	0.03 ℃	153.15	19.65
Na^+	1.13 MHz/decade	7.35 kHz	0.01 decade	88.17	9.89
pH	0.88 MHz/pH at [K⁺] = 0.1 mM	15.28 kHz	0.02 pH	173.29	12.76
K^+	0.79 MHz/decade	9.10 kHz	0.01 decade	116.61	9.46
NH_4^+	0.80 MHz/decade at [Glu] = 25 μM	20.80 kHz	0.03 decade	260.60	6.57
Glucose	0.28 MHz/μM	12.53 kHz	4.55 μM	170.81	7.29

Fig. S26. Performance metrics of the third-order EP system for biomarker monitoring. (A)–(F) Frequency shifts as functions of biomarkers (T, Na^+, pH, K^+, NH_4^+, and Glucose). Each recording is 200 s for evaluating frequency fluctuation. The insets zoom in the frequency fluctuation at the initial states (i.e., red boxes). The red arrows indicate the absolute frequency fluctuation, equal to twice the Allan deviation with a sampling time of 0.5 s. (G) Summary of performance metrics for biomarker detection. The *Q*-factors are extracted from the experimental results in figs. S20, S25, and S32, calculated as the ratio of the central resonance frequency to the bandwidth between the frequencies where the response is 3 dB above the dip minimum. The SNRs were determined as the ratio of the minimal experimentally achieved frequency shift induced by a parameter change (highlighted in the red box) to the absolute Allan deviation.

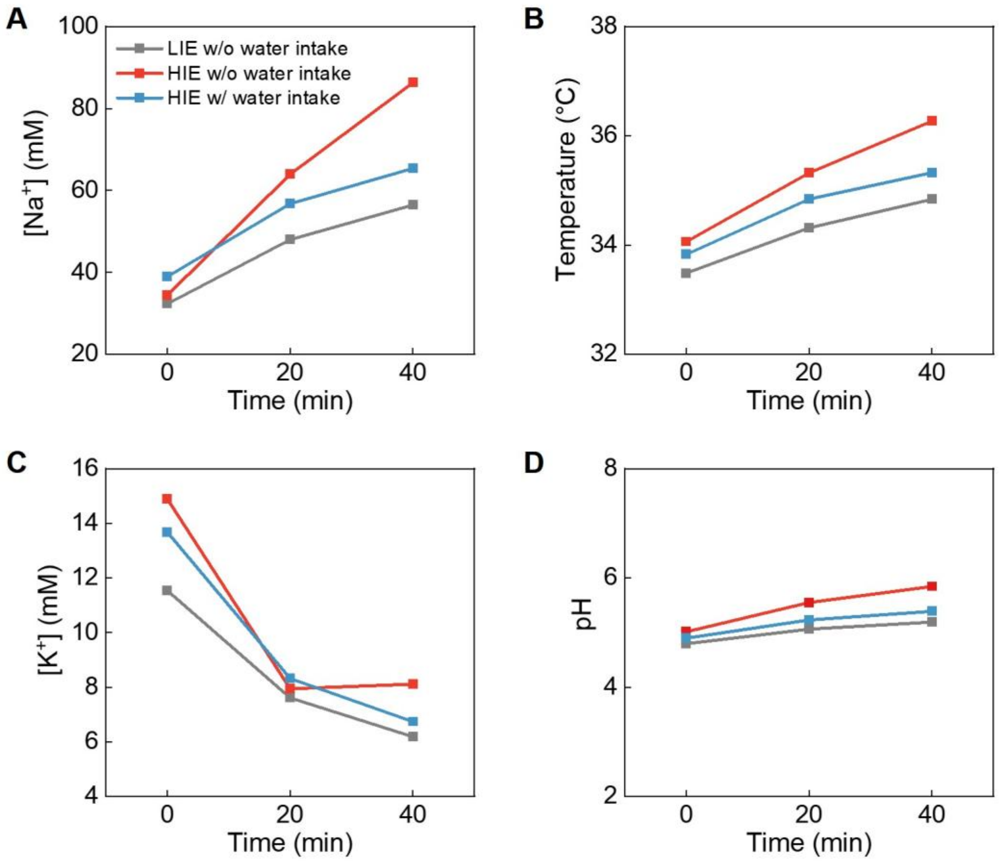

Fig. S27. Skin temperature, sweat [Na^+], [K^+], and pH during 40-min LIE, high-intensity exercise (HIE) without (w/o) water intake, and HIE with (w/) water intake.

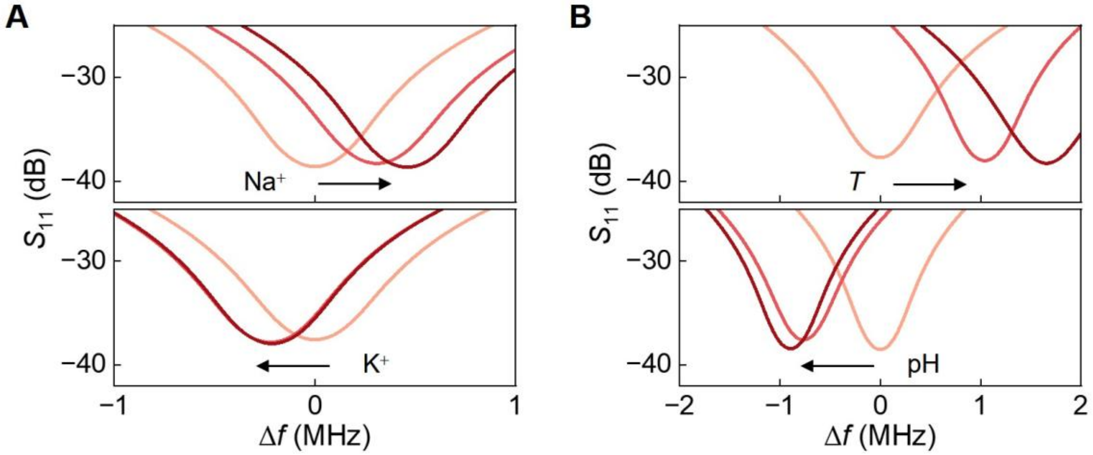

Fig. S28. Resonant frequency shifts during 40-min high-intensity exercise (LIE) without water intake. The frequency shifts indicate changes in [Na^+], [K^+], temperature (*T*), and pH.

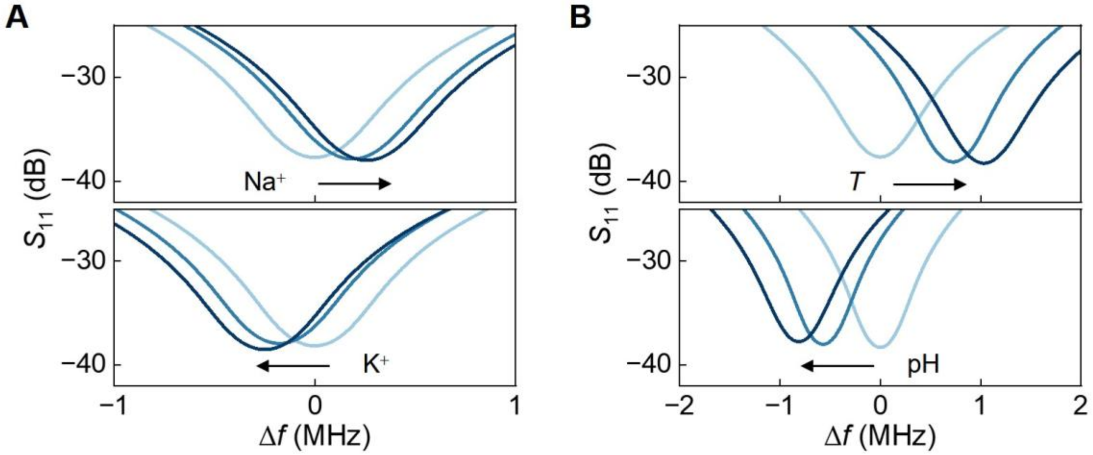

Fig. S29. Resonant frequency shifts during 40-min high-intensity exercise (LIE) with water intake (120 ml per 5 min). The frequency shifts indicate changes in [Na^+], [K^+], temperature (T), and pH.

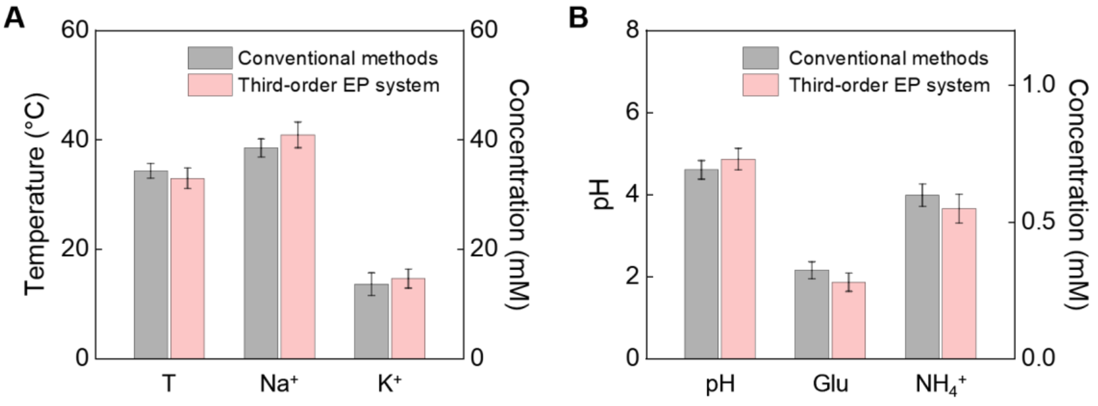

Fig. S30. Comparison of the results measured with the third-order EP sensing system and conventional methods. The skin temperature was measured using a commercial thermometer. The ion concentrations were determined using commercially available assay kits, and the glucose concentrations were validated using high-performance liquid chromatography (HPLC). The results from two methods are comparable, proving the validity of the third-order EP system. Error bars represent the standard deviations of three individual samples.

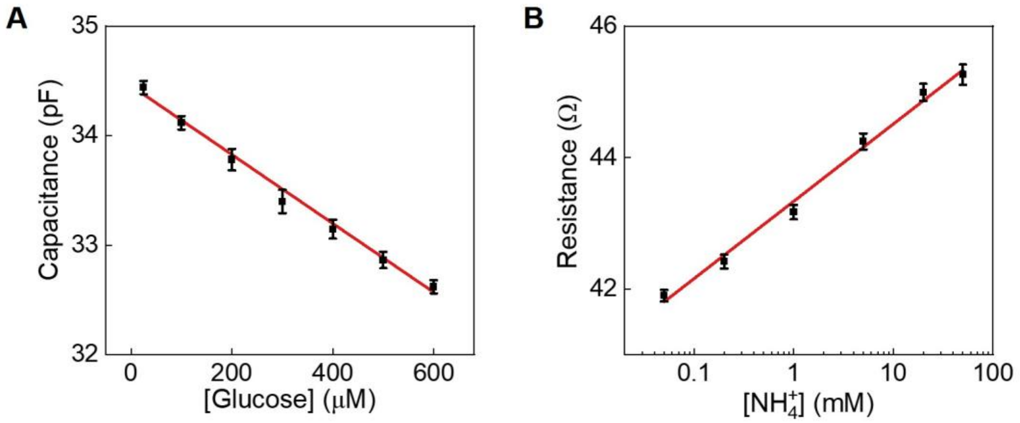

Fig. S31. Transducers employed for glucose and NH_4^+ monitoring. (A) Equivalent capacitance C_3 of the sensing resonator as a function of glucose level. (B) Equivalent resistance R_3 of the sensing resonator as a function of NH_4^+ concentration. The resistance considers the conductive traces, with details provided in fig. S1.

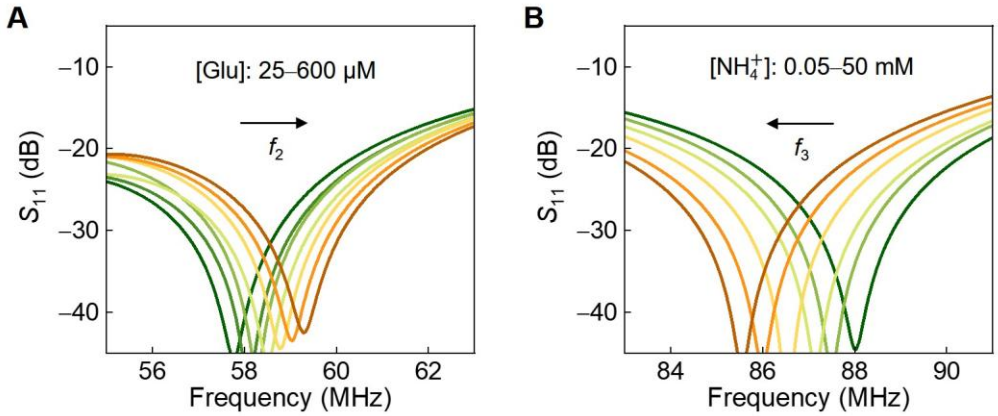

Fig. S32. Shifts in the second and third resonant frequencies with [Glu] (A) and [NH_4^+] (B). In (A), the NH_4^+ concentration is fixed at 0.05 mM and in (B), the glucose level is fixed at 25 μM.

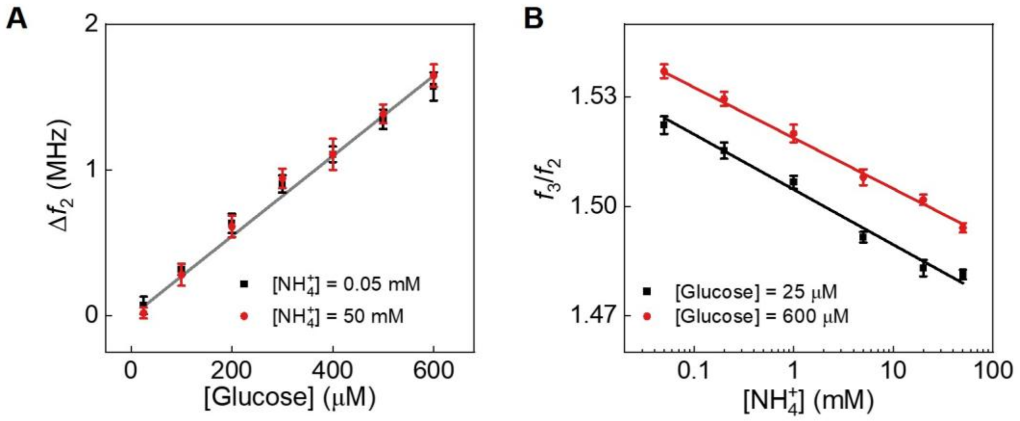

Fig. S33. Monitoring of glucose and NH_4^+ concentration. (A) The shift in the second resonant frequency f_2 with respect to glucose, independent of NH_4^+ concentration. (B) The frequency ratio f_3/f_2 as a function of NH_4^+ concentration.

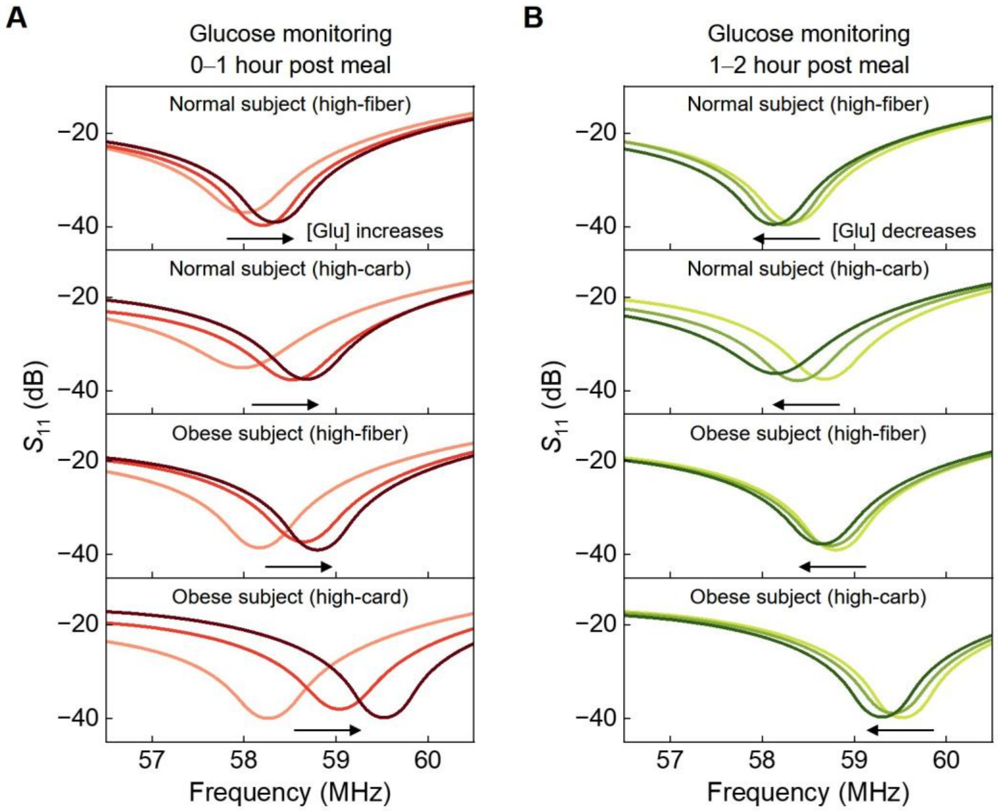

Fig. S34. Shifts in the second resonant frequency in the 0–1 hour (A) and 1–2 hour (B) post the first meal. The frequency shifts directly indicate changes in glucose levels.

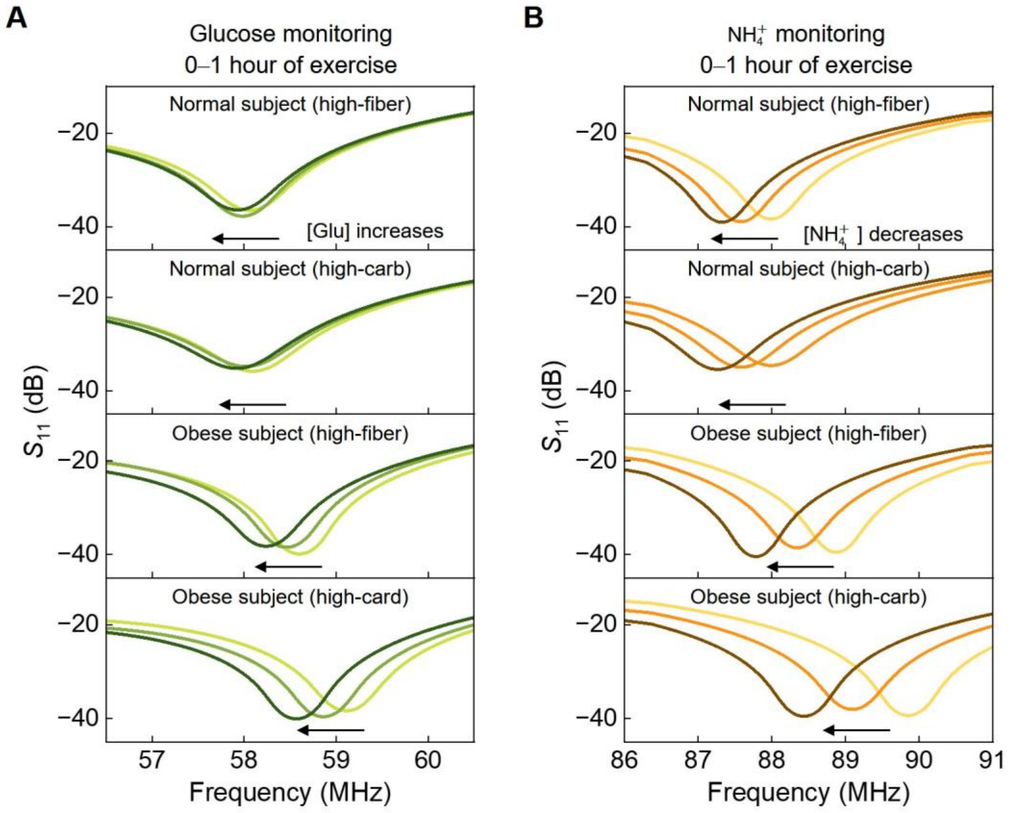

Fig. S35. Shifts in the second (A) and third (B) resonant frequency during the 0–1 hour of exercise. The frequency shifts indicate changes in glucose and NH_4^+ concentrations, respectively.

Table S1 Comparisons of the high-order EP system with existing digital wireless sensing systems

Ref.	Rigid[*] area (cm^2)	Power supply	Sensitivity boosting method	Sensing capacity[#] (parameters)	Materials	Flexi-bility	Stretch-ability
**Bluetooth-based wireless sensing systems**							
(*58*)	~4	Battery	Amplifier	1 (Strain)	Copper and FR4	No	No
(*12*)	~17	FTENG[‖]	Amplifier	2 (pH, Na^+)	Copper and PI[‖]	Yes	No
(*59*)	~2	Battery	Amplifier	2 (Sweat rate, conductivity)	Copper and PI	Yes	No
(*17*)	~3	Battery	Amplifier	3 (Phenylalanine, chloride, sweat rate)	Copper and PI	Yes	No
(*14*)	~16	Battery	Amplifier	3 (Na^+, K^+, Ca^{2+})	Copper and FR4	No	No
(*60*)	~2	Battery	Amplifier	2 (Pressure, temperature)	Copper, PI, and elastomer	Yes	No
(*61*)	~5	Battery	Amplifier	3 (Cortisol, pH, temperature)	Copper and PI	Yes	No
(*10*)	~3	Battery	Amplifier	2 (Two-site pressure)	Copper and PI	Yes	No
(*48*)	~3	Battery	Amplifier	4 (Oestradiol, pH, temperature, ionic strength)	Copper and PI	Yes	No
(*42*)	~20	Battery	Amplifier	6 (Glucose, lactate, AA[‖], UA[‖], Na^+, K^+)	Copper and FR4	No	No
(*11*)	~11	Battery	Amplifier	5 (Glucose, lactate, temperature, Na^+, K^+)	Copper, FR4, and PDMS[‖]	Yes	No
(*2*)	~13	Battery	Amplifier	9 (Glucose, lactate, UA, Na^+, K^+, NH_4^+, GSR[‖], pulse, temperature)	Copper and PI	Yes	No
**Challenges**	➢ Require external power supply or battery (causing maintenance issues)<br>➢ Large rigid component area (inevitably consisting of numerous rigid components such as Bluetooth module, microcontroller, multiplexer, and analog-to-digital converter)<br>➢ Rely on complicated circuit design (i.e., amplifiers) for boosting sensing performance<br>➢ Poor wearing comfort and non-stretchable (mismatch in Young's modulus between human skin and PI or FR4 substrates)						
**NFC[‖]-based wireless sensing systems**							
(*62*)	~0.8	None	None	1 (Staphylococcus aureus)	Copper and PI	Yes	No
(*63*)	~0.2	None	None	1 (Matrix metalloproteinase-9)	AgNF[‖]-AgNW[‖] hybrid and elastomer	Yes	Yes (30%)

(*64*)	~0.2	None	None	1 (Cortisol)	AgNF-AgNW hybrid and elastomer	Yes	Yes (30%)
(*65*)	~0.2	None	None	1 (Strain)	AgNF-AgNW hybrid and elastomer	Yes	Yes (30%)
(*66*)	~1	None	None	2 (Temperature, impedance)	Copper and PI	Yes	No
(*67*)	~0.2	None	None	1 (Glucose)	Copper, PI, and PDMS	Yes	Yes (~30%)
(*7*)	~1	None	None	1 (Ultraviolet)	Copper and PI	Yes	No
(*68*)	~0.4	None	None	1 (Pressure)	Copper and PI (circuit), LM[ǁ] and SEBS[ǁ] (coil)	Yes	Partial
(*69*)	~3	None	Amplifier	2 (Temperature, humidity)	Copper and PI (circuit), MXene and PDMS (coil)	Yes	Partial
**Challenges**	➢ Unavoidable use of rigid NFC chips<br>➢ Limited sensing capacity (only detecting one or two parameters)<br>➢ Limited sensing performance, i.e., low sensitivity<br>➢ Non-stretchable or limited stretchability (unable to achieve fully stretchable devices due to the use of NFC chips and limitations of materials)						
**This work**	**~0.05**	**None**	**High-order EP**	**2 (Glucose, NH_4^+)**	**PLMC[ǁ] and porous PU[ǁ]**	**Yes**	**Yes (300%)**
	**~0.1**			**4 (Temperature, Na^+, K^+, pH)**			
	➢ Fully passive and battery-free<br>➢ Enhanced sensitivity by frequency bifurcation near EP, not adding circuit complexity<br>➢ Multiplexed sensing capacity enabled by high-order EP, allowing dual-parameter sensing with a single resonator<br>➢ Skin-compliance and excellent stretchability (i.e., superior wearing comfort), enabled by simple circuit design and characteristics of nanomaterials (PLMC and PU)						

[*]Rigid area implies the area of rigid components (e.g., chips, amplifiers, and resistors) and does not include batteries.
[#]Sensing capacity represents the number of parameters that the sensor can monitor simultaneously.
[ǁ]Abbreviations: FTENG, freestanding triboelectric nanogenerator; PI, polyimide; AA, ascorbic acid; UA, uric acid; PDMS, polydimethylsiloxane; GSR, galvanic skin response; NFC, near-filed communication; AgNF; silver nanofiber; AgNW, silver nanowire; LM, liquid metal; SEBS, styrene-ethylene-butylene-styrene; PLMC, porous liquid metal composite; PU, polyurethane; EP, exceptional point.

Table S2 Comparisons of the high-order EP system with existing analog wireless sensing systems

Ref.	Rigid area (cm^2)	Power supply	Sensitivity boosting method	Sensing capacity (parameters)	Materials	Flexi -bility	Stretch -ability
			**"LC[‖]"-based wireless sensing systems**				
(*70*)	0	None	Chemical tuning	1 (Ammonia)	Copper and PI	Yes	No
(*71*)	0	None	None	1 (Strain)	Conductive textile and cloth	Yes	Yes (30%)
(*4*)	0	None	None	1 (Strain)	Silver composite and SEBS	Yes	Yes (50%)
(*72*)	0	None	SAW[‖]	1 (One of strain, Na^+, UV[‖])	Gold, PI, and PDMS	Yes	Yes (10%)
(*73*)	0	None	Empty core dielectric	1 (Strain)	Conductive fibre and PDMS	Yes	Yes (30%)
(*21*)	0	None	Enlarged sensing area	2 (Temperature, humidity)	Silver and tissue paper	Yes	No
(*19*)	~0.15	None	None	3 (Temperature, pH, cortisol)	Copper and PET[‖]	Yes	Yes (30%)
(*18*)	~0.05	None	None	1 (One of Na^+, K^+, H^+, Ca^{2+}, Glucose, serotonin)	Copper and PET	Yes	Yes (30%)
	~0.15			3 (Na^+, K^+, H^+)			
(*5*)	~0.15	None	None	3 (Na^+, NH_4^+, H^+)	PSPN[‖] and porous PU	Yes	Yes (100%)
	~0.1			2 (Glucose, Alcohol)			
(*8*)	0	None	Immuno crosslinks	4 (Temperature, SARS-CoV-2[‖], H1N1, RSV[‖])	Copper and PI	Yes	No
**Challenges**	➢ Limited sensing capacity (only detecting a single parameter by using an "LC" resonator and multi-parameter monitoring replying on multiple resonator tanks)<br>➢ Limited sensitivity (sensitivity enhancement relying on material or structure innovation and system-level sensitivity remaining poor)<br>➢ Non-stretchable or limited stretchability (circuit malfunction under large strain)						
			**EP[‖]-based wireless sensing systems**				
(*74*)	~4	None	EP	1 (Humidity or distance)	Copper and FR-4	No	No
(*75*)	~0.2	None	EP	1 (Intracranial pressure)	Copper and PI	Yes	No
(*76*)	~4	None	EP	1 (Humidity)	Copper and FR-4	No	No
(*77*)	~6	None	EP	1 (Temperature)	Copper and FR-4	No	No
(*25*)	~4	None	Generalized EP	1 (Pressure)	Copper and FR-4	No	No
(*78*)	~15	None	Hybrid EP	1 (Temperature)	Copper and FR-4	No	No

(*33*)	~0.02	None	EP-locked reader	1 (distance)	Copper	No	No
(*55*)	~6	None	Divergent EP	1 (Resistance or capacitance)	Copper and FR-4	No	No
(*79*)	~0.02	None	Stochastic EP	1 (Distance)	Conductive textile and cloth	Yes	Yes (~30%)
**Challenges**	➢ Limited sensing capacity (only detecting a single parameter)<br>➢ Relying on rigid printed circuit board, leading to poor wearing comfort<br>➢ Non-stretchable or limited stretchability (rare work integrating EP sensor with soft, stretchable materials)						
**This work**	**~0.05**	**None**	**High-order EP**	**2 (Glucose, NH_4^+)**	**PLMC and porous PU**	**Yes**	**Yes (300%)**
	**~0.1**			**4 (Temperature, Na^+, K^+, pH)**			
	➢ Fully passive and battery-free<br>➢ Enhanced sensitivity by frequency bifurcation near EP, not adding circuit complexity<br>➢ Multiplexed sensing capacity enabled by high-order EP, allowing dual-parameter sensing with a single resonator<br>➢ Skin-compliance and excellent stretchability (i.e., superior wearing comfort), enabled by simple circuit design and characteristics of nanomaterials (PLMC and PU)						

‖Abbreviations: LC, inductor-capacitor; SAW, surface acoustic wave; UV, ultraviolet; PET, Polyethylene terephthalate; EP, exceptional point; SARS-CoV-2, severe acute respiratory syndrome coronavirus 2; RSV, respiratory syncytial virus; PSPN, porous silver nanowire nano-composite.

REFERENCES AND NOTES

1. H. U. Chung, B. H. Kim, J. Y. Lee, J. Lee, Z. Xie, E. M. Ibler, K. Lee, A. Banks, J. Y. Jeong, J. Kim, C. Ogle, D. Grande, Y. Yu, H. Jang, P. Assem, D. Ryu, J. W. Kwak, M. Namkoong, J. B. Park, Y. Lee, D. H. Kim, A. Ryu, J. Jeong, K. You, B. Ji, Z. Liu, Q. Huo, X. Feng, Y. Deng, Y. Xu, K.-I. Jang, J. Kim, Y. Zhang, R. Ghaffari, C. M. Rand, M. Schau, A. Hamvas, D. E. Weese-Mayer, Y. Huang, S. M. Lee, C. H. Lee, N. R. Shanbhag, A. S. Paller, S. Xu, J. A. Rogers, Binodal, wireless epidermal electronic systems with in-sensor analytics for neonatal intensive care. *Science* **363**, eaau0780 (2019).

2. C. Xu, Y. Song, J. R. Sempionatto, S. A. Solomon, Y. Yu, H. Y. Y. Nyein, R. Y. Tay, J. Li, W. Heng, J. Min, A. Lao, T. K. Hsiai, J. A. Sumner, W. Gao, A physicochemical-sensing electronic skin for stress response monitoring. *Nat. Electron.* **7**, 168–179 (2024).

3. S. Kwon, H. S. Kim, K. Kwon, H. Kim, Y. S. Kim, S. H. Lee, Y.-T. Kwon, J.-W. Jeong, L. M. Trotti, A. Duarte, W.-H. Yeo, At-home wireless sleep monitoring patches for the clinical assessment of sleep quality and sleep apnea. *Sci. Adv.* **9**, eadg9671 (2023).

4. S. Niu, N. Matsuhisa, L. Beker, J. Li, S. Wang, J. Wang, Y. Jiang, X. Yan, Y. Yun, W. Burnett, A. S. Y. Poon, J. B.-H. Tok, X. Chen, Z. Bao, A wireless body area sensor network based on stretchable passive tags. *Nat. Electron.* **2**, 361–368 (2019).

5. Y. Xu, Z. Ye, G. Zhao, Q. Fei, Z. Chen, J. Li, M. Yang, Y. Ren, B. Berigan, Y. Ling, X. Qian, L. Shi, I. Ozden, J. Xie, W. Gao, P.-Y. Chen, Z. Yan, Phase-separated porous nanocomposite with ultralow percolation threshold for wireless bioelectronics. *Nat. Nanotechnol.* **19**, 1158–1167 (2024).

6. S. Li, H. Wang, W. Ma, L. Qiu, K. Xia, Y. Zhang, H. Lu, M. Zhu, X. Liang, X.-E. Wu, H. Liang, Y. Zhang, Monitoring blood pressure and cardiac function without positioning via a deep learning–assisted strain sensor array. *Sci. Adv.* **9**, eadh0615 (2023).

7. E. O. Polat, G. Mercier, I. Nikitskiy, E. Puma, T. Galan, S. Gupta, M. Montagut, J. J. Piqueras, M. Bouwens, T. Durduran, G. Konstantatos, S. Goossens, F. Koppens, Flexible graphene photodetectors for wearable fitness monitoring. *Sci. Adv.* **5**, eaaw7846 (2019).

8. X. Li, R. Sun, J. Pan, Z. Shi, Z. An, C. Dai, J. Lv, G. Liu, H. Liang, J. Liu, Y. Lu, F. Zhang, Q. Liu, Rapid and on-site wireless immunoassay of respiratory virus aerosols via hydrogel-modulated resonators. *Nat. Commun.* **15**, 4035 (2024).

9. M. Kang, H. Jeong, S.-W. Park, J. Hong, H. Lee, Y. Chae, S. Yang, J.-H. Ahn, Wireless graphene-based thermal patch for obtaining temperature distribution and performing thermography. *Sci. Adv.* **8**, eabm6693 (2022).

10. J. Li, H. Jia, J. Zhou, X. Huang, L. Xu, S. Jia, Z. Gao, K. Yao, D. Li, B. Zhang, Y. Liu, Y. Huang, Y. Hu, G. Zhao, Z. Xu, J. Li, C. K. Yiu, Y. Gao, M. Wu, Y. Jiao, Q. Zhang, X. Tai, R. H. Chan, Y. Zhang, X. Ma, X. Yu, Thin, soft, wearable system for continuous wireless monitoring of artery blood pressure. *Nat. Commun.* **14**, 5009 (2023).

11. W. Gao, S. Emaminejad, H. Y. Y. Nyein, S. Challa, K. Chen, A. Peck, H. M. Fahad, H. Ota, H. Shiraki, D. Kiriya, D.-H. Lien, G. A. Brooks, R. W. Davis, A. Javey, Fully integrated wearable sensor arrays for multiplexed in situ perspiration analysis. *Nature* **529**, 509–514 (2016).

12. Y. Song, J. Min, Y. Yu, H. Wang, Y. Yang, H. Zhang, W. Gao, Wireless battery-free wearable sweat sensor powered by human motion. *Sci. Adv.* **6**, eaay9842 (2020).

13. L. Kong, W. Li, T. Zhang, H. Ma, Y. Cao, K. Wang, Y. Zhou, A. Shamim, L. Zheng, X. Wang, W. Huang, Wireless technologies in flexible and wearable Sensing: From materials design, system integration to applications. *Adv. Mater.* **36**, 2400333 (2024).

14. X. Cai, R.-Z. Xia, Z.-H. Liu, H.-H. Dai, Y.-H. Zhao, S.-H. Chen, M. Yang, P.-H. Li, X.-J. Huang, Fully integrated multiplexed wristwatch for real-time monitoring of electrolyte ions in sweat. *ACS Nano* **18**, 12808–12819 (2024).

15. S. Olenik, H. S. Lee, F. Güder, The future of near-field communication-based wireless sensing. *Nat. Rev. Mater.* **6**, 286–288 (2021).

16. A. J. Bandodkar, P. Gutruf, J. Choi, K. Lee, Y. Sekine, J. T. Reeder, W. J. Jeang, A. J. Aranyosi, S. P. Lee, J. B. Model, R. Ghaffari, C.-J. Su, J. P. Leshock, T. Ray, A. Verrillo, K.

Thomas, V. Krishnamurthi, S. Han, J. Kim, S. Krishnan, T. Hang, J. A. Rogers, Battery-free, skin-interfaced microfluidic/electronic systems for simultaneous electrochemical, colorimetric, and volumetric analysis of sweat. *Sci. Adv.* **5**, eaav3294 (2019).

17. B. Zhong, X. Qin, H. Xu, L. Liu, L. Li, Z. Li, L. Cao, Z. Lou, J. A. Jackman, N.-J. Cho, L. Wang, Interindividual- and blood-correlated sweat phenylalanine multimodal analytical biochips for tracking exercise metabolism. *Nat. Commun.* **15**, 624 (2024).

18. T.-L. Liu, Y. Dong, S. Chen, J. Zhou, Z. Ma, J. Li, Battery-free, tuning circuit–inspired wireless sensor systems for detection of multiple biomarkers in bodily fluids. *Sci. Adv.* **8**, eabo7049 (2022).

19. Y. Dong, T.-L. Liu, S. Chen, P. Nithianandam, K. Matar, J. Li, A "Two-Part" resonance circuit based detachable sweat patch for noninvasive biochemical and biophysical sensing. *Adv. Funct. Mater.* **33**, 2210136 (2023).

20. A. R. Carr, Y. H. Patel, C. R. Neff, S. Charkhabi, N. E. Kallmyer, H. F. Angus, N. F. Reuel, Sweat monitoring beneath garments using passive, wireless resonant sensors interfaced with laser-ablated microfluidics. *NPJ Digit. Med.* **3**, 1–9 (2020).

21. W. Lv, Y. Zhang, H. Luo, Q. Xu, W. Quan, J. Yang, M. Zeng, N. Hu, Z. Yang, Wide remote-range and accurate wireless LC temperature–humidity sensor enabled by efficient mutual interference mitigation. *ACS Sens.* **8**, 4531–4541 (2023).

22. G. E. Bonacchini, F. G. Omenetto, Flexible and fully printed passive RF resonators for contact-less solution sensing. *Adv. Funct. Mater.* **34**, 2314853 (2024).

23. M.-A. Miri, A. Alù, Exceptional points in optics and photonics. *Science* **363**, eaar7709 (2019).

24. R. El-Ganainy, K. G. Makris, M. Khajavikhan, Z. H. Musslimani, S. Rotter, D. N. Christodoulides, Non-Hermitian physics and PT symmetry. *Nat. Phys.* **14**, 11–19 (2018).

25. P.-Y. Chen, M. Sakhdari, M. Hajizadegan, Q. Cui, M. M.-C. Cheng, R. El-Ganainy, A. Alù, Generalized parity–time symmetry condition for enhanced sensor telemetry. *Nat. Electron.* **1**, 297–304 (2018).

26. M. C. Rechtsman, Optical sensing gets exceptional. *Nature* **548**, 161–162 (2017).

27. M. Farhat, M. Yang, Z. Ye, P.-Y. Chen, PT-symmetric absorber-laser enables electromagnetic sensors with unprecedented sensitivity. *ACS Photonics* **7**, 2080–2088 (2020).

28. R. Fleury, D. Sounas, A. Alù, An invisible acoustic sensor based on parity-time symmetry. *Nat. Commun.* **6**, 5905 (2015).

29. W. Chen, Ş. Kaya Özdemir, G. Zhao, J. Wiersig, L. Yang, Exceptional points enhance sensing in an optical microcavity. *Nature* **548**, 192–196 (2017).

30. Z. Xiao, H. Li, T. Kottos, A. Alù, Enhanced sensing and nondegraded thermal noise performance based on PT-symmetric electronic circuits with a sixth-order exceptional point. *Phys. Rev. Lett.* **123**, 213901 (2019).

31. H. Hodaei, A. U. Hassan, S. Wittek, H. Garcia-Gracia, R. El-Ganainy, D. N. Christodoulides, M. Khajavikhan, Enhanced sensitivity at higher-order exceptional points. *Nature* **548**, 187–191 (2017).

32. P.-Y. Chen, R. El-Ganainy, Exceptional points enhance wireless readout. *Nat. Electron.* **2**, 323–324 (2019).

33. Z. Dong, Z. Li, F. Yang, C.-W. Qiu, J. S. Ho, Sensitive readout of implantable microsensors using a wireless system locked to an exceptional point. *Nat. Electron.* **2**, 335–342 (2019).

34. M. Hajizadegan, M. Sakhdari, S. Liao, P. Chen, High-sensitivity wireless displacement sensing enabled by PT-symmetric telemetry. *IEEE Trans. Antennas Propag.* **67**, 3445–3449 (2019).

35. Y. Xu, Y. Su, X. Xu, B. Arends, G. Zhao, D. N. Ackerman, H. Huang, S. P. Reid, J. L. Santarpia, C. Kim, Z. Chen, S. Mahmoud, Y. Ling, A. Brown, Q. Chen, G. Huang, J. Xie, Z. Yan, Porous liquid metal–elastomer composites with high leakage resistance and antimicrobial property for skin-interfaced bioelectronics. *Sci. Adv.* **9**, eadf0575 (2023).

36. R. Ma, B. Kang, S. Cho, M. Choi, S. Baik, Extraordinarily high conductivity of stretchable fibers of polyurethane and silver nanoflowers. *ACS Nano* **9**, 10876–10886 (2015).

37. Y. Ohm, C. Pan, M. J. Ford, X. Huang, J. Liao, C. Majidi, An electrically conductive silver–polyacrylamide–alginate hydrogel composite for soft electronics. *Nat. Electron.* **4**, 185–192 (2021).

38. D. Son, J. Kang, O. Vardoulis, Y. Kim, N. Matsuhisa, J. Y. Oh, J. W. To, J. Mun, T. Katsumata, Y. Liu, A. F. McGuire, M. Krason, F. Molina-Lopez, J. Ham, U. Kraft, Y. Lee, Y. Yun, J. B.-H. Tok, Z. Bao, An integrated self-healable electronic skin system fabricated via dynamic reconstruction of a nanostructured conducting network. *Nat. Nanotechnol.* **13**, 1057–1065 (2018).

39. R. Ye, H. Xu, C. Wan, S. Peng, L. Wang, H. Xu, Z. P. Aguilar, Y. Xiong, Z. Zeng, H. Wei, Antibacterial activity and mechanism of action of ε-poly-l-lysine. *Biochem. Biophys. Res. Commun.* **439**, 148–153 (2013).

40. Y.-Q. Li, Q. Han, J.-L. Feng, W.-L. Tian, H.-Z. Mo, Antibacterial characteristics and mechanisms of ε-poly-lysine against Escherichia coli and Staphylococcus aureus. *Food Control* **43**, 22–27 (2014).

41. K. Kwon, J. U. Kim, Y. Deng, S. R. Krishnan, J. Choi, H. Jang, K. Lee, C.-J. Su, I. Yoo, Y. Wu, L. Lipschultz, J.-H. Kim, T. S. Chung, D. Wu, Y. Park, T. Kim, R. Ghaffari, S. Lee, Y. Huang, J. A. Rogers, An on-skin platform for wireless monitoring of flow rate, cumulative loss and temperature of sweat in real time. *Nat. Electron.* **4**, 302–312 (2021).

42. W. He, C. Wang, H. Wang, M. Jian, W. Lu, X. Liang, X. Zhang, F. Yang, Y. Zhang, Integrated textile sensor patch for real-time and multiplex sweat analysis. *Sci. Adv.* **5**, eaax0649 (2019).

43. J. Hiltunen, Continuous sweat monitoring on the go. *Nat. Electron.* **6**, 557–558 (2023).

44. J. Tu, J. Min, Y. Song, C. Xu, J. Li, J. Moore, J. Hanson, E. Hu, T. Parimon, T.-Y. Wang, E. Davoodi, T.-F. Chou, P. Chen, J. J. Hsu, H. B. Rossiter, W. Gao, A wireless patch for the monitoring of C-reactive protein in sweat. *Nat. Biomed. Eng.* **7**, 1293–1306 (2023).

45. J. Choi, R. Ghaffari, L. B. Baker, J. A. Rogers, Skin-interfaced systems for sweat collection and analytics. *Sci. Adv.* **4**, eaar3921 (2018).

46. M. Bariya, H. Y. Y. Nyein, A. Javey, Wearable sweat sensors. *Nat. Electron.* **1**, 160–171 (2018).

47. Y. Liu, X. Li, H. Yang, P. Zhang, P. Wang, Y. Sun, F. Yang, W. Liu, Y. Li, Y. Tian, S. Qian, S. Chen, H. Cheng, X. Wang, Skin-interfaced superhydrophobic insensible sweat sensors for evaluating body thermoregulation and skin barrier functions. *ACS Nano* **17**, 5588–5599 (2023).

48. C. Ye, M. Wang, J. Min, R. Y. Tay, H. Lukas, J. R. Sempionatto, J. Li, C. Xu, W. Gao, A wearable aptamer nanobiosensor for non-invasive female hormone monitoring. *Nat. Nanotechnol.* **19**, 330–337 (2024).

49. D. S. Yang, R. Ghaffari, J. A. Rogers, Sweat as a diagnostic biofluid. *Science* **379**, 760–761 (2023).

50. U. Mogera, H. Guo, M. Namkoong, M. S. Rahman, T. Nguyen, L. Tian, Wearable plasmonic paper–based microfluidics for continuous sweat analysis. *Sci. Adv.* **8**, eabn1736 (2022).

51. F. Lorestani, X. Zhang, A. M. Abdullah, X. Xin, Y. Liu, M. M. Rahman, M. A. S. Biswas, B. Li, A. Dutta, Z. Niu, S. Das, S. Barai, K. Wang, H. Cheng, A highly sensitive and long-term stable wearable patch for continuous analysis of biomarkers in sweat. *Adv. Funct. Mater.* **33**, 2306117 (2023).

52. N. Brasier, J. R. Sempionatto, S. Bourke, G. Havenith, D. Schaffarczyk, J. Goldhahn, C. Lüscher, W. Gao, Towards on-skin analysis of sweat for managing disorders of substance abuse. *Nat. Biomed. Eng.* **8**, 925–929 (2024).

53. L. Tai, W. Gao, M. Chao, M. Bariya, Q. P. Ngo, Z. Shahpar, H. Y. Y. Nyein, H. Park, J. Sun, Y. Jung, E. Wu, H. M. Fahad, D. Lien, H. Ota, G. Cho, A. Javey, Methylxanthine drug monitoring with wearable sweat sensors. *Adv.Mater.* **30**, e1707442 (2018).

54. L.-C. Tai, T. S. Liaw, Y. Lin, H. Y. Y. Nyein, M. Bariya, W. Ji, M. Hettick, C. Zhao, J. Zhao, L. Hou, Z. Yuan, Z. Fan, A. Javey, Wearable sweat band for noninvasive levodopa monitoring. *Nano Lett.* **19**, 6346–6351 (2019).

55. M. Sakhdari, M. Hajizadegan, Q. Zhong, D. N. Christodoulides, R. El-Ganainy, P.-Y. Chen, Experimental observation of PT symmetry breaking near divergent exceptional points. *Phys. Rev. Lett.* **123**, 193901 (2019).

56. M. Sakhdari, Z. Ye, M. Farhat, P.-Y. Chen, Generalized theory of PT-symmetric radio-frequency systems with divergent exceptional points. *IEEE Trans. Antennas Propag.* **70**, 9396–9405 (2022).

57. G. Zhao, Y. Ling, Y. Su, Z. Chen, C. J. Mathai, O. Emeje, A. Brown, D. R. Alla, J. Huang, C. Kim, Q. Chen, X. He, D. Stalla, Y. Xu, Z. Chen, P.-Y. Chen, S. Gangopadhyay, J. Xie, Z. Yan, Laser-scribed conductive, photoactive transition metal oxide on soft elastomers for Janus on-skin electronics and soft actuators. *Sci. Adv.* **8**, eabp9734 (2022).

58. A. Abramson, C. T. Chan, Y. Khan, A. Mermin-Bunnell, N. Matsuhisa, R. Fong, R. Shad, W. Hiesinger, P. Mallick, S. S. Gambhir, Z. Bao, A flexible electronic strain sensor for the real-time monitoring of tumor regression. *Sci. Adv.* **8**, eabn6550 (2022).

59. S. Kim, S. Park, J. Choi, W. Hwang, S. Kim, I.-S. Choi, H. Yi, R. Kwak, An epifluidic electronic patch with spiking sweat clearance for event-driven perspiration monitoring. *Nat. Commun.* **13**, 6705 (2022).

60. Y. Park, K. Kwon, S. S. Kwak, D. S. Yang, J. W. Kwak, H. Luan, T. S. Chung, K. S. Chun, J. U. Kim, H. Jang, H. Ryu, H. Jeong, S. M. Won, Y. J. Kang, M. Zhang, D. Pontes, B. R. Kampmeier, S. H. Seo, J. Zhao, I. Jung, Y. Huang, S. Xu, J. A. Rogers, Wireless, skin-interfaced sensors for compression therapy. *Sci. Adv.* **6**, eabe1655 (2020).

61. B. Wang, C. Zhao, Z. Wang, K.-A. Yang, X. Cheng, W. Liu, W. Yu, S. Lin, Y. Zhao, K. M. Cheung, H. Lin, H. Hojaiji, P. S. Weiss, M. N. Stojanović, A. J. Tomiyama, A. M. Andrews, S. Emaminejad, Wearable aptamer-field-effect transistor sensing system for noninvasive cortisol monitoring. *Sci. Adv.* **8**, eabk0967 (2022).

62. Z. Xiong, S. Achavananthadith, S. Lian, L. E. Madden, Z. X. Ong, W. Chua, V. Kalidasan, Z. Li, Z. Liu, P. Singh, H. Yang, S. P. Heussler, S. M. P. Kalaiselvi, M. B. H. Breese, H. Yao, Y. Gao, K. Sanmugam, B. C. K. Tee, P.-Y. Chen, W. Loke, C. T. Lim, G. S. H. Chiang, B. Y. Tan, H. Li, D. L. Becker, J. S. Ho, A wireless and battery-free wound infection sensor based on DNA hydrogel. *Sci. Adv.* **7**, eabj1617 (2021).

63. J. Jang, J. Kim, H. Shin, Y.-G. Park, B. J. Joo, H. Seo, J. Won, D. W. Kim, C. Y. Lee, H. K. Kim, J.-U. Park, Smart contact lens and transparent heat patch for remote monitoring and therapy of chronic ocular surface inflammation using mobiles. *Sci. Adv.* **7**, eabf7194 (2021).

64. M. Ku, J. Kim, J.-E. Won, W. Kang, Y.-G. Park, J. Park, J.-H. Lee, J. Cheon, H. H. Lee, J.-U. Park, Smart, soft contact lens for wireless immunosensing of cortisol. *Sci. Adv.* **6**, eabb2891 (2020).

65. J. Kim, J. Park, Y.-G. Park, E. Cha, M. Ku, H. S. An, K.-P. Lee, M.-I. Huh, J. Kim, T.-S. Kim, D. W. Kim, H. K. Kim, J.-U. Park, A soft and transparent contact lens for the wireless quantitative monitoring of intraocular pressure. *Nat. Biomed. Eng.* **5**, 772–782 (2021).

66. Y. Jiang, A. A. Trotsyuk, S. Niu, D. Henn, K. Chen, C.-C. Shih, M. R. Larson, A. M. Mermin-Bunnell, S. Mittal, J.-C. Lai, A. Saberi, E. Beard, S. Jing, D. Zhong, S. R. Steele, K. Sun, T. Jain, E. Zhao, C. R. Neimeth, W. G. Viana, J. Tang, D. Sivaraj, J. Padmanabhan, M. Rodrigues, D. P. Perrault, A. Chattopadhyay, Z. N. Maan, M. C. Leeolou, C. A. Bonham, S. H. Kwon, H. C. Kussie, K. S. Fischer, G. Gurusankar, K. Liang, K. Zhang, R. Nag, M. P. Snyder, M. Januszyk, G. C. Gurtner, Z. Bao, Wireless, closed-loop, smart bandage with integrated sensors and stimulators for advanced wound care and accelerated healing. *Nat. Biotechnol.* **41**, 652–662 (2023).

67. W. Park, H. Seo, J. Kim, Y.-M. Hong, H. Song, B. J. Joo, S. Kim, E. Kim, C.-G. Yae, J. Kim, J. Jin, J. Kim, Y. Lee, J. Kim, H. K. Kim, J.-U. Park, In-depth correlation analysis between tear glucose and blood glucose using a wireless smart contact lens. *Nat. Commun.* **15**, 2828 (2024).

68. J. Choi, C. Han, S. Cho, K. Kim, J. Ahn, D. Del Orbe, I. Cho, Z.-J. Zhao, Y. S. Oh, H. Hong, S. S. Kim, I. Park, Customizable, conformal, and stretchable 3D electronics via predistorted pattern generation and thermoforming. *Sci. Adv.* **7**, eabj0694 (2021).

69. Y. Shao, L. Wei, X. Wu, C. Jiang, Y. Yao, B. Peng, H. Chen, J. Huangfu, Y. Ying, C. J. Zhang, J. Ping, Room-temperature high-precision printing of flexible wireless electronics based on MXene inks. *Nat. Commun.* **13**, 3223 (2022).

70. W. Lv, J. Yang, Q. Xu, J. A.-A. Mehrez, J. Shi, W. Quan, H. Luo, M. Zeng, N. Hu, T. Wang, H. Wei, Z. Yang, Wide-range and high-accuracy wireless sensor with self-humidity compensation for real-time ammonia monitoring. *Nat. Commun.* **15**, 6936 (2024).

71. V. Galli, S. K. Sailapu, T. J. Cuthbert, C. Ahmadizadeh, B. C. Hannigan, C. Menon, Passive and wireless all-textile wearable sensor system. *Adv. Sci.* **10**, 2206665 (2023).

72. Y. Kim, J. M. Suh, J. Shin, Y. Liu, H. Yeon, K. Qiao, H. S. Kum, C. Kim, H. E. Lee, C. Choi, H. Kim, D. Lee, J. Lee, J.-H. Kang, B.-I. Park, S. Kang, J. Kim, S. Kim, J. A. Perozek, K. Wang, Y. Park, K. Kishen, L. Kong, T. Palacios, J. Park, M.-C. Park, H. Kim, Y. S. Lee, K. Lee, S.-H. Bae, W. Kong, J. Han, J. Kim, Chip-less wireless electronic skins by remote epitaxial freestanding compound semiconductors. *Science* **377**, 859–864 (2022).

73. J. Lee, S. J. Ihle, G. S. Pellegrino, H. Kim, J. Yea, C.-Y. Jeon, H.-C. Son, C. Jin, D. Eberli, F. Schmid, B. L. Zambrano, A. F. Renz, C. Forró, H. Choi, K.-I. Jang, R. Küng, J. Vörös, Stretchable and suturable fibre sensors for wireless monitoring of connective tissue strain. *Nat. Electron.* **4**, 291–301 (2021).

74. B.-B. Zhou, W.-J. Deng, L.-F. Wang, L. Dong, Q.-A. Huang, Enhancing the remote distance of LC passive wireless sensors by parity-time symmetry breaking. *Phys. Rev. Applied* **13**, 064022 (2020).

75. M. Yang, Z. Ye, N. Alsaab, M. Farhat, P.-Y. Chen, In-vitro demonstration of ultra-reliable, wireless and batteryless implanted intracranial sensors operated on loci of exceptional points. *IEEE Trans. Biomed. Circuits Syst.* **16**, 287–295 (2022).

76. B.-B. Zhou, W.-D. Liu, L. Dong, The sensitivity of PT-symmetric LC wireless sensors around an exceptional point. *Appl. Phys. Lett.* **123**, 164103 (2023).

77. M. Yang, Z. Ye, M. Farhat, P.-Y. Chen, Ultrarobust wireless interrogation for sensors and transducers: A non-hermitian telemetry technique. *IEEE Trans. Instrum. Meas.* **70**, 1–9 (2021).

78. Y. Hao, C. Ding, P. Wu, G. Wang, Y. Cheng, Bidirectional wireless sensing based on coexistent PT and anti-PT symmetries. *IEEE Trans. Instrum. Meas.* **73**, 1–12 (2024).

79. Z. Li, C. Li, Z. Xiong, G. Xu, Y. R. Wang, X. Tian, X. Yang, Z. Liu, Q. Zeng, R. Lin, Y. Li, J. K. W. Lee, J. S. Ho, C.-W. Qiu, Stochastic exceptional points for noise-assisted sensing. *Phys. Rev. Lett.* **130**, 227201 (2023).
